# Association between household composition and severe COVID-19 outcomes in older people by ethnicity: an observational cohort study using the OpenSAFELY platform

**DOI:** 10.1093/ije/dyac158

**Published:** 2022-08-13

**Authors:** Kevin Wing, Daniel J Grint, Rohini Mathur, Hamish P Gibbs, George Hickman, Emily Nightingale, Anna Schultze, Harriet Forbes, Vahé Nafilyan, Krishnan Bhaskaran, Elizabeth Williamson, Thomas House, Lorenzo Pellis, Emily Herrett, Nileesa Gautam, Helen J Curtis, Christopher T Rentsch, Angel Y S Wong, Brian MacKenna, Amir Mehrkar, Seb Bacon, Ian J Douglas, Stephen J W Evans, Laurie Tomlinson, Ben Goldacre, Rosalind M Eggo

**Affiliations:** Faculty of Epidemiology and Population Health, London School of Hygiene & Tropical Medicine, London, UK; Faculty of Epidemiology and Population Health, London School of Hygiene & Tropical Medicine, London, UK; Faculty of Epidemiology and Population Health, London School of Hygiene & Tropical Medicine, London, UK; Department of Geography, University College London, London, UK; The DataLab, University of Oxford Nuffield Department of Primary Care Health Sciences, Oxford, UK; Faculty of Epidemiology and Population Health, London School of Hygiene & Tropical Medicine, London, UK; Faculty of Epidemiology and Population Health, London School of Hygiene & Tropical Medicine, London, UK; Population Health Sciences, University of Bristol, Bristol, UK; Health Modelling Hub, Office of National Statistics, Newport, UK; Faculty of Epidemiology and Population Health, London School of Hygiene & Tropical Medicine, London, UK; Faculty of Epidemiology and Population Health, London School of Hygiene & Tropical Medicine, London, UK; Department of Mathematics, University of Manchester, Manchester, UK; Department of Mathematics, University of Manchester, Manchester, UK; Faculty of Epidemiology and Population Health, London School of Hygiene & Tropical Medicine, London, UK; Faculty of Epidemiology and Population Health, London School of Hygiene & Tropical Medicine, London, UK; Aetion Inc, Boston, USA; The DataLab, University of Oxford Nuffield Department of Primary Care Health Sciences, Oxford, UK; Faculty of Epidemiology and Population Health, London School of Hygiene & Tropical Medicine, London, UK; Faculty of Epidemiology and Population Health, London School of Hygiene & Tropical Medicine, London, UK; The DataLab, University of Oxford Nuffield Department of Primary Care Health Sciences, Oxford, UK; The DataLab, University of Oxford Nuffield Department of Primary Care Health Sciences, Oxford, UK; The DataLab, University of Oxford Nuffield Department of Primary Care Health Sciences, Oxford, UK; Faculty of Epidemiology and Population Health, London School of Hygiene & Tropical Medicine, London, UK; Faculty of Epidemiology and Population Health, London School of Hygiene & Tropical Medicine, London, UK; Faculty of Epidemiology and Population Health, London School of Hygiene & Tropical Medicine, London, UK; The DataLab, University of Oxford Nuffield Department of Primary Care Health Sciences, Oxford, UK; The DataLab, University of Oxford Nuffield Department of Primary Care Health Sciences, Oxford, UK

**Keywords:** COVID-19, household, ethnicity, multigenerational, older people, deprivation, comorbidities, population-level, OpenSAFELY

## Abstract

**Background:**

Ethnic differences in the risk of severe COVID-19 may be linked to household composition. We quantified the association between household composition and risk of severe COVID-19 by ethnicity for older individuals.

**Methods:**

With the approval of NHS England, we analysed ethnic differences in the association between household composition and severe COVID-19 in people aged 67 or over in England. We defined households by number of age-based generations living together, and used multivariable Cox regression stratified by location and wave of the pandemic and accounted for age, sex, comorbidities, smoking, obesity, housing density and deprivation. We included 2 692 223 people over 67 years in Wave 1 (1 February 2020–31 August 2020) and 2 731 427 in Wave 2 (1 September 2020–31 January 2021).

**Results:**

Multigenerational living was associated with increased risk of severe COVID-19 for White and South Asian older people in both waves [e.g. Wave 2, 67+ living with three other generations vs 67+-year-olds only: White hazard ratio (HR) 1.61 95% CI 1.38–1.87, South Asian HR 1.76 95% CI 1.48–2.10], with a trend for increased risks of severe COVID-19 with increasing generations in Wave 2. There was also an increased risk of severe COVID-19 in Wave 1 associated with living alone for White (HR 1.35 95% CI 1.30–1.41), South Asian (HR 1.47 95% CI 1.18–1.84) and Other (HR 1.72 95% CI 0.99–2.97) ethnicities, an effect that persisted for White older people in Wave 2.

**Conclusions:**

Both multigenerational living and living alone were associated with severe COVID-19 in older adults. Older South Asian people are over-represented within multigenerational households in England, especially in the most deprived settings, whereas a substantial proportion of White older people live alone. The number of generations in a household, number of occupants, ethnicity and deprivation status are important considerations in the continued roll-out of COVID-19 vaccination and targeting of interventions for future pandemics.

Key Messages

- This is the first study to analyse up-to-date population-level data to assess the effect of multigenerational living on severe COVID-19 by ethnicity in the UK.
- UK National Health Service primary and secondary care data from February 2020 onwards were used to study the association between household composition and severe COVID-19 in older people for Wave 1 and Wave 2 of the pandemic.
- Living with more younger generations was associated with an increased hazard of severe COVID-19 for White and South Asian older people in both waves, with a trend of increasing severe COVID-19 with increasing generations in Wave 2. Living alone also increased the hazard of severe COVID-19, particularly for White older people.
- Despite there being over 10 times the number of older White people in England than older South Asian people, nearly twice the number of South Asian older people live in multigenerational houses in the most deprived settings.
- For South Asian older people in these households, rates of severe COVID-19 during Wave 2 were higher than those for older people with multiple (established severe COVID-19 risk factor) comorbidities.
- In contrast, the majority of cases of severe COVID-19 in White people were within those living alone.

## Introduction

The composition of a household—the ages and number of its members—is a key determinant of infection risk for many infections, including COVID-19.^1–5^ Households with multiple age-based generations may be at higher risk of infection due to increased routes of household introduction, with increased contact between older adults and working-age adults of particular concern. Differences in the proportion of multigenerational households by ethnicity may be an underlying factor in the disproportionate effect that COVID-19 has had on ethnic minority groups in the UK.^1^^,^^6–11^ Analysing how multigenerational living affects the risk of severe COVID-19 in people of retirement age (over 67 in the UK) across Wave 1 and Wave 2 of the pandemic could improve our understanding of drivers of ethnic disparities in COVID-19 outcomes.

Using the OpenSAFELY platform, we sought to assess whether: (i) household composition was associated with severe COVID-19 in older people within individual ethnic groups after accounting for potential confounders; and (ii) whether any association between household composition and severe COVID-19 and other potential household-level explanatory factors differed by ethnicity.

## Methods

### Study design and population

We used linked primary care electronic health record data for 24 million people in England from the OpenSAFELY-TPP platform (see Supplementary Material, available as Supplementary data at *IJE* online). We extracted separate study populations for Waves one (1 February 2020 to 31 August 2020) and two (1 September 2020 to 31 January 2021). We selected people aged 67 years or older at the start of each wave to represent a population (i) at or over retirement age and (ii) at risk of severe COVID-19 due to their age. We wanted to assess if people of retirement age were at a differential risk of severe COVID-19 if they lived with younger generations who were more likely to be working (or attending educational or child care settings). Participants were followed up until the earliest of: the outcome of interest; deregistration from their general practice; death from any cause; or the end date for each wave.

Our pre-specified study protocol [https://github.com/opensafely/hh-classification-research/tree/master/docs] and (post hoc) deviations from the protocol (Supplementary Table S1, available as Supplementary data at *IJE* online) are available.

#### Inclusion and exclusion criteria

Participants with at least 3 months of follow-up before the study start date for each wave were included, to ensure adequate capture of baseline factors. We used a TPP-developed pseudonymized household identifier which links people living at the same address on 1 February 2020^12^ (see Supplementary Methods, available as Supplementary data at *IJE* online). We excluded people with no household identifier, households with anyone flagged as living in a care home (with care home status derived by matching addresses to Care Quality Commission data^13^), those living with more than 12 people (possible care homes or other institutions), and those living with more than four people who were all over the age of 67 (possible care homes).^13^ We also excluded people with missing sex, location (based on Middle Layer Super Output Area—MSOA) or index of multiple deprivation (indicators of poor data quality when missing).

### Exposure

We classified distinct generations as 0–17-year-olds, 18–29-year-olds, 30–66-year-olds and 67+-year-olds before assigning each 67+-year-old in our study to one of the following five household composition categories:

- sixty-seven+ living alone: 67+-year-old living alone (single occupancy household);
- multiple 67+-year-olds: 67+-year-old living with up to three other 67+-year-olds (reference category);
- one other generation: 67+-year-old(s) living with people from just ONE other younger generation;
- two other generations: 67+-year-old(s) living with people from TWO other younger generations;
- three other generations: 67+-year-old(s) living with people from all THREE other younger generations.

The primary exposure was the household composition where each 67+-year-old was resident on 1 February 2020.

### Outcomes

The primary outcome was ‘severe COVID-19’ defined as COVID-19 related hospital admission or death between 1 February 2020 and 31 August 2020 (for Wave 1), and 1 September 2020 and 31 January 2021 (for Wave 2):

- hospital admission with COVID-19: a COVID-19 ICD-10 code for confirmed (U07.1) or suspected (U07.2) COVID-19 in the primary diagnosis field in Secondary Use Service (SUS) data;
- COVID-19-related death: a COVID-19 ICD-10 code for confirmed or suspected COVID-19 anywhere on the death certificate.

We also analysed each of the above outcomes separately, and included a secondary outcome of ‘non-COVID-19 death’ (death from any other cause on the death certificate) to assess specificity of results.

### Covariates

#### Ethnicity

We investigated the effect of household composition within GP-recorded census ethnicity categories of White, South Asian, Black, Mixed and Other. Detailed census categories (Supplementary Table S2, available as Supplementary data at *IJE* online) were analysed for any ethnicity where household composition was associated with severe COVID-19.

#### Other covariates

We included categorical age in years (67–69, 70–74, 75–79, 80–84, 85+), sex, body mass index categories (kg/m^2^) (underweight, normal, overweight, obese I, obese II, obese III), index of multiple deprivation quintiles (from 1, most affluent, to 5, most deprived), geographical region defined by Upper Tier Local Authority (UTLA), smoking status (current, former, never), housing density (ONS Rural/Urban classification in categories of urban major/minor conurbation, urban city and town, rural town, rural village) and number of comorbidities shown to be associated with poor COVID-19 outcomes (0, 1 or 2+)^14^ (see Supplementary Tables S3 and S4, available as Supplementary data at *IJE* online).

### Statistical analysis

#### Descriptive analysis

Participant characteristics at baseline were summarized by household composition category separately for Wave 1 and for Wave 2.

#### Household composition and severe COVID-19/non-COVID-19 death by ethnicity

We used multivariable Cox proportional hazards regression with robust standard errors to account for clustering by household in order to estimate differences by household composition in the hazard of severe COVID-19 and non-COVID-19 death. All models were stratified by UTLA region to account for region-specific variation in infection rates over time, ^15^ and separate analyses were performed for Wave 1 and Wave 2. An interaction between household composition and ethnicity was included in all models, with all household composition results reported by ethnicity. We hypothesized that associations between other covariates and severe COVID-19 would vary by ethnicity, so performed likelihood ratio tests for interaction (LRT) between each covariate and ethnicity (including interactions as appropriate in our final models). Where deprivation or housing density interacted with ethnicity, we also present results stratified by ethnicity for these variables.

We assessed the proportional hazards assumption by testing for a zero slope in the scaled Schoenfeld residuals and through graphical inspection of plots of the Schoenfeld residuals against time.

#### Analysis of the impact of household size (number of occupants)

In our main analysis of household composition we decided a priori not to adjust for household size (see page 2 of Supplementary Methods and Table S5, available as Supplementary data at *IJE* online). Instead, to assess how the hazard of severe COVID varied by number of occupants within categories of household composition and vice versa, we cross-tabulated results from a combined household composition-household size exposure variable (Supplementary Table S6, available as Supplementary data at *IJE* online).

#### Absolute rates of severe COVID-19 and non-COVID-19 death by household characteristics

Rates of all outcomes were reported by ethnicity and: (i) household composition; (ii) household size; (iii) any other household-level explanatory variables found to differ by ethnicity; and (iv) those with two or more comorbidities (for comparison).

#### Sensitivity analyses

We tested the impact of including a 5-year ‘buffer’ between the 67+ year old generation and the next youngest generation (to avoid a 67+-year-old living with a 61–66-year-old being considered multigenerational). We assessed the impact of only including people who lived in households with 100% TPP coverage (i.e. all adults in the household were registered with TPP—see Supplementary Methods). Our main analysis was a complete ethnicity records analysis—we performed a sensitivity analysis applying multiple imputation to account for missing ethnicity (10 imputations). In our main analysis, people with missing body mass index were assumed to be normal weight, and those with missing smoking data were assumed to be never smokers (on the assumption that obesity and smoking would likely be recorded if present); a complete records sensitivity analysis for body mass index (BMI) and smoking was performed.

#### Software and reproducibility

This analysis was delivered through the OpenSAFELY platform—see Supplementary Material for details.

#### Patient and public involvement

We have developed a publicly available website [https://opensafely.org/] through which we invite any patient or member of the public to contact us regarding this study or the broader OpenSAFELY project.

#### Role of the funding source

The funders of the study had no role in study design, data collection, data analysis, data interpretation or writing of the report.

## Results

### Descriptive results

From a total of 23 696 832 individuals in the OpenSAFELY database on 1 February, 2020, there were 2 692 223 people aged 67 or over (referred to as: ‘67+’) at the beginning of Wave 1 who met the selection criteria (Supplementary Figure S1, available as Supplementary data at *IJE* online). Of these, 1 109 443 (41.2%) lived with other 67+, 920 670 (34.2%) lived alone, 526 037 (19.5%) lived with one other younger generation, 113 553 (4.2%) lived with two other younger generations and 22 540 (0.8%) lived with three other younger generations (Table 1). The final Wave 2 cohort was slightly larger (2 731 427 people), with no change in relative proportions of household compositions (Table 1).

**Table 1:** Baseline characteristics of cohort of 67+-year-olds, by categories of generational household composition during the first and second waves of the pandemic in England

	Wave 1 (1 February–31 August 2020)						Wave 2 (1 September–31 January 2021)					
		Household composition (number of generations in household)						Household composition (number of generations in household)				
Characteristic	Total (*n *= 2 692 223)	Multiple 67+-year-olds (*n* = 1 109 443)	67+ living alone (*n* = 920 670)	67+ & 1 (*n* = 526 037)	67+ & 2 (*n* = 113 533)	67+ & 3 (*n* = 22 540)	Total (*n* = 2 731 427)	Multiple 67+-year-olds (*n* = 1 143 558)	67+ living alone (*n* = 914 039)	67+ & 1 (*n* = 534 276)	67+ & 2 (*n* = 116 278)	67+ & 3 (n = 23 276)
Sex												
F	1 450 088 (53.9)	560 567 (50.5)	586 899 (63.7)	237 493 (45.1)	53 200 (46.9)	11 929 (52.9)	1 470 941 (53.9)	580 492 (50.8)	581 276 (63.6)	242 588 (45.4)	54 212 (46.6)	12 373 (53.2)
M	1242135 (46.1)	548 876 (49.5)	333 771 (36.3)	288 544 (54.9)	60 333 (53.1)	10 611 (47.1)	1 260 486 (46.1)	563 066 (49.2)	332 763 (36.4)	291 688 (54.6)	62 066 (53.4)	10 903 (46.8)
Age (years)												
Mean (SD)	75.8 (6.7)	75.5 (5.8)	77.8 (7.5)	73.5 (6.2)	73.7 (6.2)	74.5 (6.5)	75.9 (6.7)	75.6 (5.9)	77.9 (7.5)	73.6 (6.2)	73.6 (6.2)	74.5 (6.5)
Median (IQR)	74.0 (70.0-80.0)	74.0 (71.0-79.0)	77.0 (72.0-83.0)	72.0 (69.0-77.0)	72.0 (69.0-77.0)	73.0 (69.0-79.0)	74.0 (71.0-80.0)	75.0 (71.0-79.0)	77.0 (72.0-83.0)	72.0 (69.0-77.0)	72.0 (69.0-77.0)	73.0 (69.0-79.0)
Categorical												
67–69	503 732 (18.7)	163 996 (14.8)	128 739 (14.0)	168 840 (32.1)	35 857 (31.6)	6300 (28.0)	505 939 (18.5)	164 493 (14.4)	127 802 (14.0)	170 305 (31.9)	36 954 (31.8)	6385 (27.4)
70–74	856 665 (31.8)	393 791 (35.5)	237 702 (25.8)	180 272 (34.3)	37 902 (33.4)	6998 (31.0)	869 008 (31.8)	402 170 (35.2)	236 997 (25.9)	183 442 (34.3)	39 056 (33.6)	7343 (31.5)
75–79	592 679 (22.0)	286 581 (25.8)	194 667 (21.1)	87 617 (16.7)	19 582 (17.2)	4232 (18.8)	605 755 (22.2)	297 690 (26.0)	193 972 (21.2)	89 779 (16.8)	19 880 (17.1)	4434 (19.0)
80–84	403 923 (15.0)	170 999 (15.4)	167 516 (18.2)	50 654 (9.6)	11 820 (10.4)	2934 (13.0)	408 023 (14.9)	177 839 (15.6)	163 864 (17.9)	51 432 (9.6)	11 875 (10.2)	3013 (12.9)
85+	335 224 (12.5)	94 076 (8.5)	192 046 (20.9)	38 654 (7.3)	8372 (7.4)	2076 (9.2)	342 702 (12.5)	101 366 (8.9)	191 404 (20.9)	39 318 (7.4)	8513 (7.3)	2101 (9.0)
Ethnicity												
White	2 548 262 (94.7)	1 083 552 (97.7)	887 343 (96.4)	482 696 (91.8)	82 362 (72.5)	12 309 (54.6)	2 583 394 (94.6)	1 116 533 (97.6)	880 434 (96.3)	489 303 (91.6)	84 368 (72.6)	12 756 (54.8)
South Asian	87 132 (3.2)	15 264 (1.4)	14 674 (1.6)	26 557 (5.0)	22 650 (20.0)	7987 (35.4)	89 653 (3.3)	15 969 (1.4)	14 745 (1.6)	27 605 (5.2)	23 136 (19.9)	8198 (35.2)
Black	26 478 (1.0)	4019 (0.4)	9399 (1.0)	7888 (1.5)	4017 (3.5)	1155 (5.1)	26 948 (1.0)	4132 (0.4)	9462 (1.0)	8050 (1.5)	4119 (3.5)	1185 (5.1)
Mixed	9394 (0.3)	2027 (0.2)	3129 (0.3)	2711 (0.5)	1209 (1.1)	318 (1.4)	9696 (0.4)	2117 (0.2)	3188 (0.3)	2832 (0.5)	1234 (1.1)	325 (1.4)
Other	20 957 (0.8)	4581 (0.4)	6125 (0.7)	6185 (1.2)	3295 (2.9)	771 (3.4)	21 736 (0.8)	4807 (0.4)	6210 (0.7)	6486 (1.2)	3421 (2.9)	812 (3.5)
BMI category												
Underweight	58 508 (2.2)	19 078 (1.7)	28 043 (3.0)	9027 (1.7)	1899 (1.7)	461 (2.0)	32 136 (1.2)	11 011 (1.0)	15 141 (1.7)	4801 (0.9)	978 (0.8)	205 (0.9)
Normal^a^	956 951 (35.5)	395 450 (35.6)	350 630 (38.1)	169 195 (32.2)	35 184 (31.0)	6492 (28.8)	1 784 671 (65.3)	748 250 (65.4)	609 405 (66.7)	338 803 (63.4)	73 784 (63.5)	14 429 (62.0)
Overweight	979 424 (36.4)	424 681 (38.3)	310 771 (33.8)	195 590 (37.2)	40 785 (35.9)	7597 (33.7)	499 221 (18.3)	220 083 (19.2)	155 953 (17.1)	98 704 (18.5)	20 539 (17.7)	3942 (16.9)
Obese I	478 907 (17.8)	191 553 (17.3)	155 410 (16.9)	102 917 (19.6)	23 784 (20.9)	5243 (23.3)	276 361 (10.1)	112 692 (9.9)	86 992 (9.5)	60 007 (11.2)	13 650 (11.7)	3020 (13.0)
Obese II	157 352 (5.8)	57 988 (5.2)	53 931 (5.9)	35 082 (6.7)	8404 (7.4)	1947 (8.6)	98 799 (3.6)	37 341 (3.3)	32 776 (3.6)	22 375 (4.2)	5112 (4.4)	1195 (5.1)
Obese III	61 081 (2.3)	20 693 (1.9)	21 885 (2.4)	14 226 (2.7)	3477 (3.1)	800 (3.5)	40 239 (1.5)	14 181 (1.2)	13 772 (1.5)	9586 (1.8)	2215 (1.9)	485 (2.1)
Smoking status												
Never^b^	1 070 550 (39.8)	443 554 (40.0)	358 348 (38.9)	204 251 (38.8)	52 581 (46.3)	11 816 (52.4)	1 089 415 (39.9)	458 986 (40.1)	355 800 (38.9)	208 636 (39.1)	53 816 (46.3)	12177 (52.3)
Former	1 408 300 (52.3)	605 090 (54.5)	473 653 (51.4)	271 542 (51.6)	49 669 (43.7)	8346 (37.0)	1 427 167 (52.2)	622 275 (54.4)	470 381 (51.5)	275 153 (51.5)	50 746 (43.6)	8612 (37.0)
Current	213 373 (7.9)	60 799 (5.5)	88 669 (9.6)	50 244 (9.6)	11 283 (9.9)	2378 (10.6)	214 845 (7.9)	62 297 (5.4)	87 858 (9.6)	50 487 (9.4)	11 716 (10.1)	2487 (10.7)
Index of multiple deprivation												
1 (affluent)	664 384 (24.7)	324 311 (29.2)	196 213 (21.3)	118 676 (22.6)	22 151 (19.5)	3033 (13.5)	676 016 (24.7)	334 864 (29.3)	195 057 (21.3)	120 245 (22.5)	22 681 (19.5)	3169 (13.6)
2	625 275 (23.2)	280 387 (25.3)	200 876 (21.8)	117 551 (22.3)	22 943 (20.2)	3518 (15.6)	645 354 (23.6)	293 400 (25.7)	203 112 (22.2)	121 387 (22.7)	23 817 (20.5)	3638 (15.6)
3	577 223 (21.4)	237 830 (21.4)	196 871 (21.4)	113 728 (21.6)	24 351 (21.4)	4443 (19.7)	581 409 (21.3)	242 636 (21.2)	194 530 (21.3)	114 770 (21.5)	24 828 (21.4)	4645 (20.0)
4	477 195 (17.7)	167 960 (15.1)	179 416 (19.5)	99 825 (19.0)	24 248 (21.4)	5746 (25.5)	476 261 (17.4)	170 310 (14.9)	175 362 (19.2)	100 189 (18.8)	24 573 (21.1)	5827 (25.0)
5 (deprived)	336834 (12.5)	94 155 (8.5)	142 899 (15.5)	74 609 (14.2)	19 424 (17.1)	5747 (25.5)	338 945 (12.4)	96 509 (8.4)	140 941 (15.4)	75 708 (14.2)	19 865 (17.1)	5922 (25.4)
Region of England												
East	631 645 (23.5)	270 459 (24.4)	205 218 (22.3)	124 028 (23.6)	26 875 (23.7)	5065 (22.5)	641 327 (23.5)	278 664 (24.4)	203 580 (22.3)	126 120 (23.6)	27 698 (23.8)	5265 (22.6)
East Midlands	499 640 (18.6)	215 911 (19.5)	162 124 (17.6)	97 962 (18.6)	19 742 (17.4)	3901 (17.3)	506 526 (18.5)	222 541 (19.5)	160 463 (17.6)	99 207 (18.6)	20 271 (17.4)	4044 (17.4)
London	119 412 (4.4)	23 076 (2.1)	43 479 (4.7)	34 018 (6.5)	15 216 (13.4)	3623 (16.1)	120 579 (4.4)	23 695 (2.1)	43 151 (4.7)	34 762 (6.5)	15 346 (13.2)	3625 (15.6)
North East	139 434 (5.2)	59 213 (5.3)	48 600 (5.3)	26 450 (5.0)	4361 (3.8)	810 (3.6)	140 938 (5.2)	60 798 (5.3)	48 048 (5.3)	26 749 (5.0)	4499 (3.9)	844 (3.6)
North West	262 220 (9.7)	108 562 (9.8)	93 956 (10.2)	50 864 (9.7)	7622 (6.7)	1216 (5.4)	265 738 (9.7)	111 821 (9.8)	93 244 (10.2)	51 585 (9.7)	7778 (6.7)	1310 (5.6)
South East	180 063 (6.7)	75 065 (6.8)	63 875 (6.9)	33 450 (6.4)	6668 (5.9)	1005 (4.5)	183 145 (6.7)	77 631 (6.8)	63 667 (7.0)	33 945 (6.4)	6846 (5.9)	1056 (4.5)
South West	408 689 (15.2)	181 212 (16.3)	139 861 (15.2)	72 401 (13.8)	13 377 (11.8)	1838 (8.2)	416 226 (15.2)	187 337 (16.4)	139 798 (15.3)	73 582 (13.8)	13 612 (11.7)	1897 (8.2)
West Midlands	94 762 (3.5)	32 525 (2.9)	34 180 (3.7)	20 904 (4.0)	5675 (5.0)	1478 (6.6)	95 434 (3.5)	33 244 (2.9)	33 661 (3.7)	21 203 (4.0)	5787 (5.0)	1539 (6.6)
Yorkshire	355 818 (13.2)	143 251 (12.9)	129 154 (14.0)	65 847 (12.5)	13 964 (12.3)	3602 (16.0)	360 97 (13.2)	147 644 (12.9)	128 220 (14.0)	67 005 (12.5)	14 409 (12.4)	3694 (15.9)
Housing density												
Conurbation												
Urban major	421 379 (15.7)	130 132 (11.7)	154 613 (16.8)	96 556 (18.4)	31 749 (28.0)	8329 (37.0)	425 489 (15.6)	133 554 (11.7)	152 870 (16.7)	98 309 (18.4)	322 66 (27.7)	8490 (36.5)
Urban minor	172 486 (6.4)	69 736 (6.3)	62 353 (6.8)	33 533 (6.4)	5792 (5.1)	1072 (4.8)	174 064 (6.4)	71 620 (6.3)	61 529 (6.7)	33 850 (6.3)	5927 (5.1)	1138 (4.9)
Urban city & town	1 350 131 (50.1)	557 061 (50.2)	470 702 (51.1)	259 693 (49.4)	52 802 (46.5)	9873 (43.8)	1 368 303 (50.1)	573 239 (50.1)	466 797 (51.1)	263 678 (49.4)	54 318 (46.7)	10 271 (44.1)
Rural												
Town	408 668 (15.2)	190 703 (17.2)	133 360 (14.5)	71 820 (13.7)	11 232 (9.9)	1553 (6.9)	414 986 (15.2)	196 876 (17.2)	132 392 (14.5)	72 628 (13.6)	11 496 (9.9)	1594 (6.8)
Village	328 247 (12.2)	157 011 (14.2)	95 247 (10.3)	62 787 (11.9)	11 542 (10.2)	1660 (7.4)	335 143 (12.3)	162 430 (14.2)	95 414 (10.4)	63 834 (11.9)	11 757 (10.1)	1708 (7.3)
Number of comorbidities												
0	1 087 692 (40.4)	480 966 (43.4)	331 798 (36.0)	222 583 (42.3)	44 714 (39.4)	7631 (33.9)	1 134 668 (41.5)	507 918 (44.4)	339 683 (37.2)	231 972 (43.4)	47 004 (40.4)	8091 (34.8)
1	847 951 (31.5)	345 372 (31.1)	293 795 (31.9)	165 060 (31.4)	36 367 (32.0)	7357 (32.6)	858 490 (31.4)	354 537 (31.0)	291 709 (31.9)	167 136 (31.3)	37 432 (32.2)	7676 (33.0)
2+	756 580 (28.1)	283 105 (25.5)	295 077 (32.1)	138 394 (26.3)	32 452 (28.6)	7552 (33.5)	738 269 (27.0)	281 103 (24.6)	282 647 (30.9)	135 168 (25.3)	31 842 (27.4)	7509 (32.3)

Data are *n* (%) unless specified.

IQR, interquartile range; BMI, Body Mass Index.

a BMI ‘normal’ includes those with missing data.

b Smoking status ‘never’ includes those with missing data.

**Figure 1:**
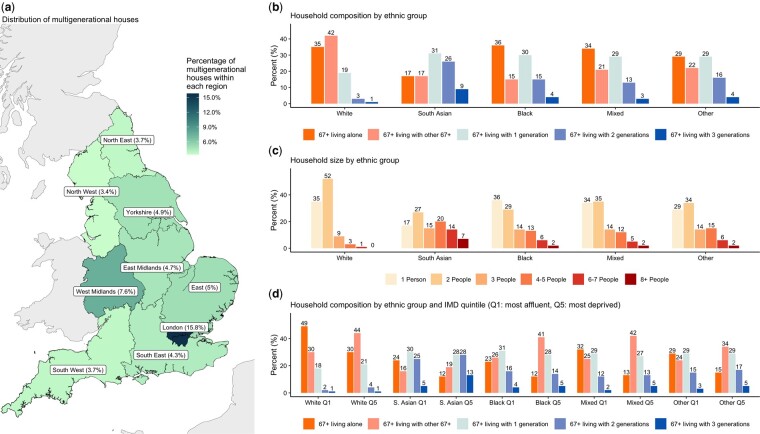
Summary population characteristics of English households by ethnicity. a] Distribution of multigenerational houses by region of England; b] age-generational household composition by ethnic group; c] household size (total number of occupants) by ethnic group; d] age-generational household composition by ethnic group and IMD quintile (Q1: most affluent, Q5: most deprived). IMD, Index of Multiple Deprivation

London had the highest proportion of 67+ living in multigenerational houses (15.8%), with a much lower proportion in the North West (3.4%) (Figure 1). Younger 67+ and those living in urban areas were more likely to live with multiple younger generations (Table 1). A notably larger proportion of South Asian (66%) and Black (49%) 67+ were living with one or more other generations than White 67+ (23%) (Figure 1). Household composition by ethnicity for Wave 2 was similar to Wave 1 (Supplementary Table S7, available as Supplementary data at *IJE* online).

### Household composition and severe COVID-19 by ethnicity

#### Wave 1

**Figure 2:**
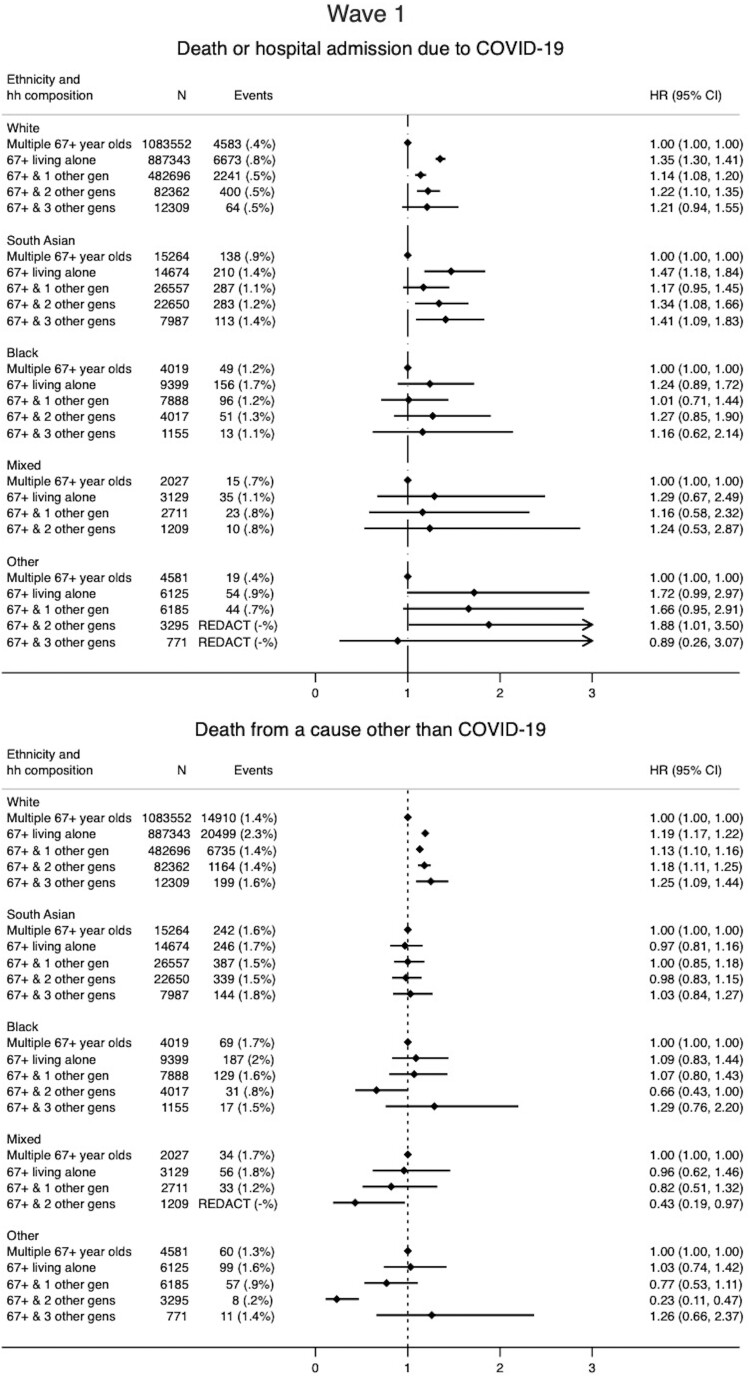
Association between household composition and (1) severe COVID-19 (death or hospitalization due to COVID-19) and (2) non-COVID-19 death by ethnicity for Wave 1 of the pandemic in England (1 February 2020 to 31 August 2020). Household composition in terms of number of distinct generations that each 67+-year-old in the study cohort is living with (considering the following distinct generations: 0–17 year olds, 18–29-year-olds, 30–66-year-olds, 67+-year-olds), i.e. 67+ & 1 = 67+year-old’s household includes one other younger generation; 67+ & 2 = 67+-year-old’s household includes two other younger generations; 67+ & 3 = 67+-year-old’s household includes three other younger generations. Models stratified by location (Upper Tier Local Authority) and adjusted for sex, number of comorbidities, categories of housing density (rural or urban setting), smoking status, socioeconomic status, and including an interaction between ethnicity and age (as well as the interaction between household composition and ethnicity presented here)

After accounting for age, sex, comorbidities, housing density, deprivation status, obesity and smoking, and including interactions between ethnicity and household composition and age (Supplementary Table S8, available as Supplementary data at *IJE* online), White 67+ living alone had an increased risk of severe COVID-19 compared with the reference group [hazard ratio (HR) 1.35 95% CI 1.30–1.41] [Figure 2 (W1)]. There was a small increase in hazard for 67+ living with any other younger generation (e.g. 67+ and two other generations: HR 1.22 95% CI 1.10–1.35) (Figure 2). For South Asian 67+, there was also an increase in hazard of severe COVID-19 in those living alone (HR 1.47 95% CI 1.18–1.84), and living with either two or three generations (e.g. three other generations HR 1.41 95% CI 1.09–1.83). People classified as ethnicity of ‘Other’ and living alone had an increased hazard of severe COVID-19 (HR 1.72 95% CI 0.99–2.97). For Black and Mixed ethnicities, wide confidence intervals limited interpretation (Figure 2), but estimates were generally consistent with an increased HR for multigenerational living and for living alone (Figure 2).

For White people, the association of household composition with non-COVID-19 death was similar to the association with severe COVID-19, with the exception of a weakened association between living alone and non-COVID-19 death (HR 1.19 95% CI 1.17–1.22). For South Asian people, associations were specific to COVID-19 (e.g. non-COVID-19 death 67+ with two other generations: HR 0.98 0.83–1.15) (Figure 2). Despite wide confidence intervals, results for other ethnicities were generally consistent with those for South Asian people.

#### Wave 2

**Figure 3:**
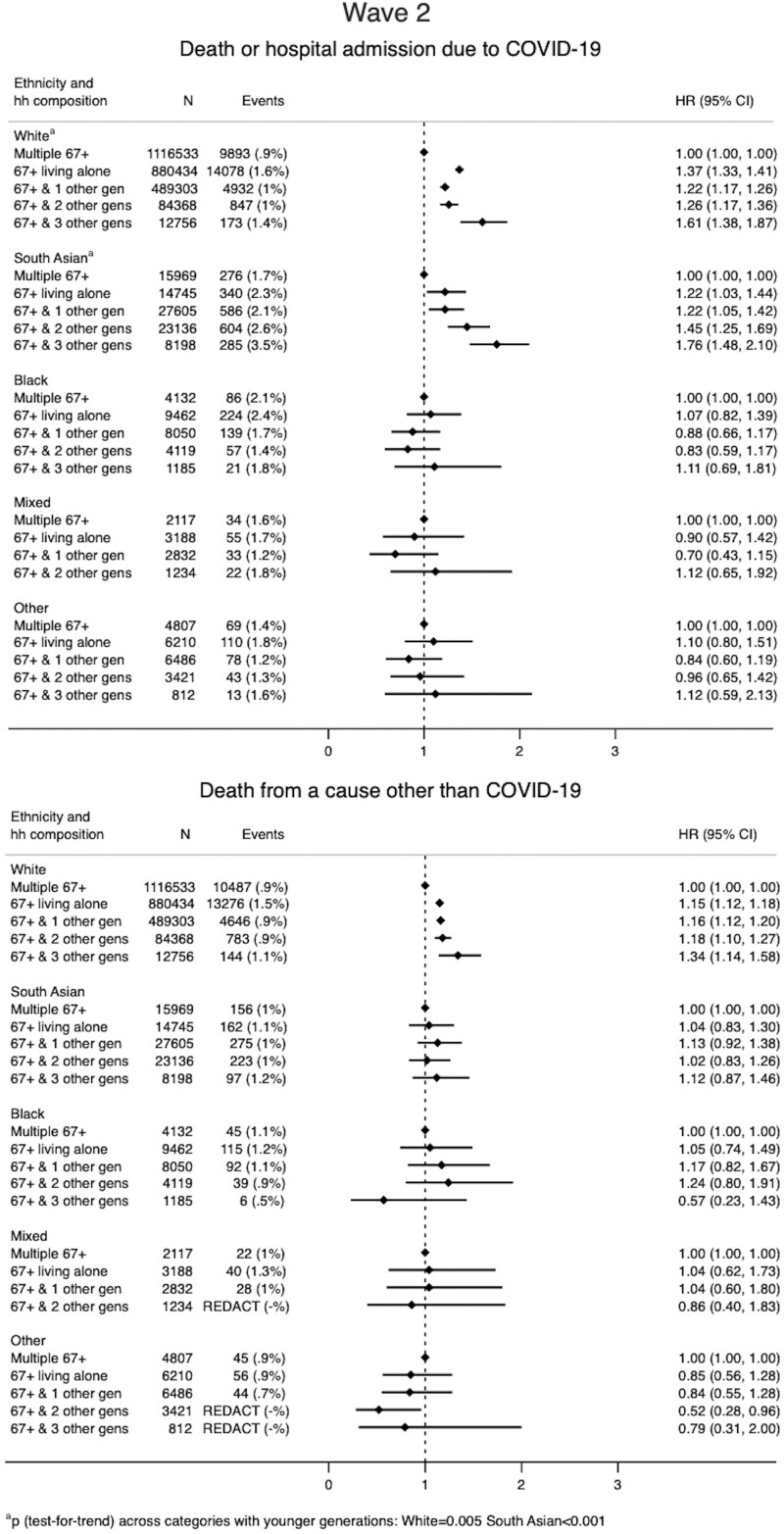
Association between household composition and (1) severe COVID-19 (death or hospitalization due to COVID-19) and (2) non-COVID-19 death by ethnicity for Wave 2 of the pandemic in England (September 2020 and 31 January 2021). Household composition in terms of number of distinct generations that each 67+-year-old in the study cohort is living with (considering the following distinct generations: 0–17-year-olds, 18–29-year-olds, 30–66-year-olds, 67+-year-olds), i.e. 67+ & 1 = 67+-year-old’s household includes one other younger generation; 67+ & 2 = 67+-year-old’s household includes two other younger generations; 67+ & 3 = 67+-year-old’s household includes three other younger generations. Models stratified on location (Upper Tier Local Authority) and adjusted for: sex, smoking, housing density and number of comorbidities, and including interactions between ethnicity and: index of multiple deprivation, age and obesity (as well as the interaction with household composition presented here)

After accounting for sex, comorbidities, housing density and smoking, and including interactions between ethnicity and: household composition, age, deprivation status and obesity (Supplementary Table S8, available as Supplementary data at *IJE* online), multigenerational living for White people in Wave 2 was associated with higher hazards of severe COVID-19 compared with Wave 1 (e.g. 67+ with three other generations Wave 2 HR 1.61 95% CI 1.38–1.87) (Figure 3) and there was evidence for an increasing hazard of severe COVID-19 with increasing number of generations (Figure 3). The increased risk of severe COVID-19 in older White people living alone in Wave 2 was similar to Wave 1 (Wave 2 HR 1.37 95% CI 1.33–1.41). For South Asian people, a similar trend was observed (Figure 3), with increases in HRs for all categories of multigenerational living compared with Wave 1 (e.g. 67+ with three other generations Wave 2 HR 1.76 95% CI 1.48–2.10) (Figure 3), whereas the association between severe COVID-19 and living alone was weakened compared with Wave 1 (Wave 2 HR 1.22 95% CI 1.03–1.44).

For South Asian people, as for Wave 1, multigenerational living was associated with severe COVID-19 but not non-COVID-19 death (Wave 2, 67+ with three other generations: non-COVID-19 death HR 1.12 95% CI 0.87–1.46) (Figure 3). For White people, risks for severe COVID-19 and non-COVID-19 death were similar during Wave 2, with the exception of those living alone, where the increased risk was higher for severe COVID-19 (severe COVID-19 HR 1.37 95% CI 1.33–1.41 vs non-COVID-death HR 1.15 95% CI 1.12–1.18). For Black and Mixed ethnicities, although wide confidence intervals limited interpretation (Figure 3), there was no evidence that increased multigenerational living was associated with an increased hazard of severe COVID-19 or non-COVID death (Figure 3).

In analysis of the South Asian ethnicity subgroups (Indian, Pakistani, Bangladeshi, Other South Asian), Indian, Pakistani and Other South Asian ethnicities were comparable and consistent with the overall effect, whereas for Bangladeshi 67+-year-olds there was no obvious pattern of increasing harm with increasing generations (although confidence intervals were wide). For the White ethnicity subgroups (British, Irish, Other White), British and Other White 67+-year-olds were driving the overall effect for Wave 2 (Supplementary Figure S3, available as Supplementary data at *IJE* online), with less evidence of a harmful association for Irish 67+-year-olds (although confidence intervals were wide).

Results for the separate severe COVID-19 outcomes (death and hospitalization) and for the analysis of the impact of household size are provided in the Supplementary Material, available as Supplementary data at *IJE* online (pages 13 and 16).

There was no evidence of deviations from the proportional hazards assumption for Wave 1 (*P* = 0.596) or Wave 2 (*P* = 0.467).

#### Other household-level variables and severe COVID-19 by ethnicity

There was strong evidence for a larger association between Index of Multiple Deprivation (IMD) and severe COVID-19 for South Asian 67+-year-olds compared with White 67+-year-olds in Wave 2 (IMD most deprived vs least deprived—White: HR 1.65 95% CI 1.58–1.72, South Asian: HR 2.46 95% CI 2.00–3.03) (Supplementary Table S10, available as Supplementary data at *IJE* online). There was weaker evidence for a larger association between IMD and severe COVID-19 for South Asian 67+-year-olds compared with Black and Other 67+-year-olds (IMD most deprived vs least deprived—Black: HR 1.76 95% CI 1.10–2.80, Other: HR 1.30 95% CI 0.88–1.93) (Supplementary Table S10). These differences were specific to severe COVID-19 (Supplementary Table S10).

#### Absolute effects

Over 13% of South Asian people lived in multigenerational households in the most deprived settings (i.e. 67+ with three generations in the 5th deprivation quintile) compared with less than 1% of White people (Table 2). This meant that despite South Asian people making up only 3.2% of the total study population (compared with 94.7% White) (Table 1), there were a larger number of South Asian 67+-year-olds (2841) than White 67+-year-olds (2377) living in the highest risk household composition arrangement. South Asian people in this group experienced nearly triple the rate of severe COVID-19 (11 802 per 100 000 person-years) than White people in this group (4293 per 100 000 person-years) (Table 2), and the rate of severe COVID-19 for South Asian people in this group was higher than in those with two or more comorbidities (9905 per 100 000 person-years) (Table 2).

**Table 2:** Rates of severe COVID by household composition, household size and deprivation status during Wave 2 for White and South Asian ethnicities (with figures for number of comorbidities included for comparison)

**Wave 2 (1 September**–**31 January 2021)**				
	*n* (%)	Events (%)	Person-years follow-up	Rate (per 100 000 person-years)
**White**				
Index of Multiple Deprivation (IMD)				
1 (least deprived)	658 568 (25.6)	5604 (18.8)	272 071	2060
2	623 364 (24.2)	6018 (20.2)	257 287	2339
3	550 578 (21.4)	6059 (20.4)	227 038	2669
4	435 765 (17.0)	6173 (20.7)	179 315	3443
5 (most deprived)	302 301 (11.8)	5919 (19.9)	123 922	4776
Generational household composition				
Multiple 67-year-olds	1 116 533 (43.2)	9893 (33.1)	461 106	2145
67+ living alone	880 434 (34.1)	14 078 (47.0)	361 731	3892
67+ & 1 other generation	489 303 (18.9)	4932 (16.5)	201 980	2442
67+ & 2 other generations	84 368 (3.3)	847 (2.8)	34 845	2431
67+ & 3 other generations	12 756 (0.5)	173 (0.6)	5257	3291
IMD and household composition				
IMD = 5, 67+ living alone	131 221 (43.4)	3132 (52.9)	53 615.4	5842
IMD = 5, 67+ & 3 other generations	2377 (0.8)	42 (0.7)	978	4293
Household size (number of occupants)				
1–2	2 245 408 (86.9)	26 269 (87.8)	925 456	2838
3–5	314 342 (12.2)	3356 (11.2)	129 712	2587
6+	23 644 (0.9)	298 (1.0)	9750	3056
IMD and household size				
IMD = 5, household size = 6+	3471 (1.1)	60 (1.0)	1428	4202
Number of comorbidities (for comparison)				
2 or more	684 355 (26.5)	17 158 (57.3)	278 833	6154
**South Asian**				
Index of Multiple Deprivation (IMD)				
1 (least deprived)	9897 (11.1)	108 (5.2)	4088	2642
2	12 422 (13.9)	214 (10.3)	5123	4177
3	19 307 (21.6)	383 (18.4)	7946	4820
4	25 963 (29.1)	653 (31.3)	10 656	6128
5 (most deprived)	21 713 (24.3)	725 (34.8)	8871	8173
Generational household composition				
Multiple 67+-year-olds	15 969 (17.8)	276 (13.2)	6572	4200
67+ living alone	14 745 (16.4)	340 (16.3)	6064	5607
67+ & 1 other generation	27 605 (30.8)	586 (28.0)	11 355	5161
67+ & 2 other generations	23 136 (25.8)	604 (28.9)	9494	6362
67+ & 3 other generations	8198 (9.1)	285 (13.6)	3345	8520
IMD and household composition				
IMD = 5, 67+ living alone	3905 (18.0)	109 (15.0)	1604	6797
IMD = 5, 67+ & 3 other generations	2841 (13.1)	136 (18.8)	1152	11 802
Household size (number of occupants)				
1–2	38 953 (43.4)	799 (38.2)	16 019	4988
3–5	31 918 (35.6)	672 (32.1)	13 133	5117
6+	18 782 (20.9)	620 (29.7)	7678	8075
IMD and household size				
IMD = 5, household size = 6+	5594 (25.8)	245 (33.8)	2271	10 790
Number of comorbidities (for comparison)				
2 or more	35 051 (39.1)	1411 (67.5)	14 246	9905

Due to the large proportion of White older people living alone (34.1%) and the relatively high rate of severe COVID-19 in this group (3892 per 100 000 person-years), nearly half (47.0%) of all severe COVID-19 cases in older White people occurred among those living alone (with only 16.3% of all severe COVID-19 cases in older South Asian people occurring among those living alone) (Table 2).

#### Sensitivity analyses

All sensitivity analyses had minimal impact on results (Supplementary Table S11, available as Supplementary data at *IJE* online).

## Discussion

### Principal findings

Multigenerational living was associated with increased risk of severe COVID-19 among older South Asian and White people in Waves 1 and 2 of the SARS-CoV-2 pandemic in England, with evidence for a trend in increasing risk of severe COVID-19 with increasing number of generations in Wave 2. Living alone was also associated with an increased risk for White and South Asian older people in both waves. For all other ethnicities, results for multigenerational living or living alone were consistent with harmful effects for Wave 1 only. For South Asian older people, results were highly specific to COVID-19 and the effect of deprivation on severe COVID-19 outcomes was greater in South Asian than in White people. Very high rates of severe COVID-19 were observed for older people in multigenerational households in the most deprived settings. Despite there being over 10 times the number of White people in England than South Asian people, nearly twice as many South Asian people live in multigenerational households in the most deprived settings compared with White people. Due to the over-representation of White people living alone, this group contributed nearly half of all cases of severe COVID-19 in White people.

### Comparison with other studies

Our findings are consistent with two studies of older people (from the UK and from Sweden) which analysed COVID-19 mortality alone^6^^,^^11^ and found increased risks associated with both multigenerational living and with living alone. Another UK study found that living with children was a risk factor for COVID-19 mortality in Wave 2,^12^ and a study in the UK Biobank found an increased risk of severe COVID-19 in larger households during combined Waves 1 and 2 (in younger people than those studied here).^16^ A number of studies have reported ethnic disparities in COVID-19 outcomes in the UK.^17–21^ Of these, none studied age-based generational household composition by ethnic group and only two studies analysed effects over both of the first two waves of the pandemic.^17^^,^^21^

Our study complements and advances these previous studies by using both up-to-date household composition and covariate data from a very large population-representative sample of England to illustrate that both living alone and living in multigenerational houses increased the risk of severe COVID-19, and that as the number of generations in a household increased, the risk of severe COVID-19 for older people increased, with effects particularly pronounced for White and South Asian older people during Wave 2. Absolute rates of severe COVID-19 in Wave 2 were very high for South Asian people living in multigenerational houses in the most deprived settings, and nearly half of all cases of severe COVID-19 in older White people were in those living alone.

### Strengths and limitations

Our study is the first to use up-to-date household and covariate information to study household composition and severe COVID-19 in England by ethnicity, and was able to analyse effects over the first two waves where lockdown restrictions differed. The key strengths of this study are the scale, detail and completeness of the underlying health record data. We could therefore assess whether there was an increasing trend for COVID-19 harms in older people living with increasing numbers of generations by ethnicity, and assess the impact of other potential household-level explanatory variables (household size and deprivation).

Our study had a number of potential weaknesses. First, 12% of our cohort who did not have a household identifier were excluded. Furthermore, for the main analysis, 22% of the cohort were not included due to missing ethnicity data, although conclusions were identical when applying multiple imputation to account for missing ethnicity. There are a number of potential issues with the linkage between TPP primary care health records and household composition data. First of all, as household occupancy was determined only once (in February 2020), we were not able to account for people moving house during the pandemic. This could mean that there was some misclassification of our main exposure (likely non-differential with respect to outcome), with people who moved house being assigned to a household composition category that may not have reflected their true household composition for some of the study period. Although it is possible that misclassification could occur between both adjacent and non-adjacent categories of household composition (which has the potential to bias results in either direction^22^), we consider that the impact of this misclassification is likely to be negligible due to: (i) low numbers of people moving due to government advice not to move during the pandemic (particularly during Wave 1^23^); and (ii) the fact that our household composition figures by ethnicity were comparable to 2011 census data (Supplementary Table S12, available as Supplementary data at *IJE* online). There may also have been some misclassification of household composition due to including some houses where not all occupants were registered at general practitioner (GP) practices using TPP software, although our sensitivity analysis including only 100% TPP households had no impact on results. Finally, there were a number of potential explanatory factors that we could not include in this analysis, such as occupation^24^ and household crowding (i.e. whether a household has fewer bedrooms than it needs to avoid undesirable sharing).^25^

### Interpretation

Our results suggest that during Wave 1 there were harmful effects of living with younger generations which were specific to COVID-19 for South Asian and the other minority ethnicities, but no different from the effect on non-COVID-19 death for White older people. People from ethnic minority groups have been shown to live in lower-quality housing than White people in England,^26^ and the impact of this on all health outcomes might be expected to be particularly severe as the number of generations in a household increases. It is notable, however, that the association between multigenerational living and severe COVID-19 outcomes in Wave 1 persisted in our analysis even after adjusting for socioeconomic status, and that any potential difference in housing quality did not also translate to an increase in non-COVID death. It may be possible that previously acknowledged general health benefits of multigenerational living may have reduced the risk of non-COVID death but not severe COVID-19 among the ethnic minority groups.^10^^,^^27^ Observed results in Wave 1 could also have been driven specifically by underlying differences in household overcrowding between White and ethnicity minority groups,^25^ as this is likely to increase the potential of older people coming into close contact with younger household members who are infected, although a previous study found that overcrowding was associated with an increased risk of both COVID-19 and non-COVID-19 death.^6^ It is also possible that strict lockdown intervention messaging was more effective at reaching White people during Wave 1,^28^ although existing evidence on this is mixed, e.g. showing that at the start of the pandemic, White people were more likely to correctly identify COVID-19 symptoms than people of other ethnicities,^29^ but providing inconsistent evidence on whether trust in government differed by ethnic group.^30^ Underlying difference in type of occupation by ethnicity may also have contributed to the observed results. For older people in minority ethnic groups, although younger household occupants could provide support, these may have been more likely to be employed in essential worker occupations (such as in hospitals, pharmacies or food shops) than White younger household occupants, leading to an increased infection risk for their older co-occupant(s).^28^,^31^

For Wave 2, increased multigenerational living had a large and specific effect on severe COVID-19 for South Asian older people, whereas results for other minority groups were no longer consistent with harm, and results for White people indicated increasing risks by increasing generations which remained similar to those for non-COVID death. UK survey results have suggested that the ability to self-isolate is lower in minority ethnic groups than in White groups, although in this analysis Black and South Asian ethnicities were grouped together^32^ and our Wave 2 severe COVID-19 results show different effects for Black compared with South Asian older people. It is possible that a greater degree of overcrowding in South Asian households than for other ethnicities may be driving Wave 2 effects,^25^ although of note is that in the analysis of the South Asian ethnicity subgroups (Supplementary Figure S3), the results for Bangladeshi households were not consistent with a harmful effect of multigenerational living, despite results from the English Housing Survey (April 2016–March 2019) indicating that Bangladeshi households had the highest percentage of overcrowding.^25^ It is likely that, as restrictions eased, there were differences by ethnicity in other key risk factors for transmission such as occupation type,^33^ inter-household mixing, religion and experiences of structural racism.^17^ This could have led to increased exposure for South Asian people at work (or in education), and also for any South Asian older people in multigenerational houses. The fact that the effect of deprivation on severe COVID-19 was greater for South Asian older people than for White (and to some extent Black and Other) people provides further confirmation that differences in COVID-19 outcomes by ethnicity cannot be explained by deprivation alone.^34^

In Wave 1 we found living alone to be associated with severe COVID-19 for White, South Asian and Other ethnicities, with the elevated risk persisting for White people in Wave 2. Living alone increases loneliness, which is an established risk factor for negative mental and physical health outcomes, suicide and all-cause mortality.^35–37^ During the UK-wide lockdowns, as loneliness intensified for those living alone^38^ one might have expected to observe an increase in both severe COVID-19 outcomes (due to people experiencing loneliness being particularly severely affected by having COVID-19) and non-COVID death (due to loneliness itself increasing all-cause mortality). In fact, for White and South Asian ethnicities, associations were notably stronger for severe COVID-19 than for non-COVID death for both waves, particularly for South Asian older people. This specificity to COVID-19 suggests that observed associations may be due to older people living alone being unable to rely on others to perform essential tasks outside the house (such as picking up medicines or buying shopping), as much as those living with other 67+-year-olds (or even other generations during Wave 1), and therefore being more likely to be infected.

Finally, although the relative effects of multigenerational living were similar across White and South Asian groups during Wave 2, the absolute effect differed substantially, with over one and a half times more people living in the highest-risk household/deprivation category (*n* = 5587 South Asian vs *n* = 3415 White). As this group experienced over twice the rate of severe COVID-19 than their White counterparts and the rate was comparable to that in people with multiple established comorbidity risk factors, it is highly likely that these household-level characteristics are contributing to previously observed imbalances in severe COVID-19 outcomes by ethnicity which particularly affect South Asian people.^17^^,^^21^. In contrast, the over-representation of White people living alone and the relatively high rate among this group means that appropriate tailoring of public health interventions could substantially reduce the numbers of severe cases among White older people in future pandemics.

## Conclusions

Multigenerational living was associated with severe COVID-19, particularly for South Asian and (to a lesser extent) White older people in both waves, with results consistent with harmful effects for other ethnicities in Wave 1 only. Living alone was also associated with severe COVID-19, an effect that persisted for White people across both waves. The established COVID-19 risk factor of deprivation has a greater effect on serious COVID-19 outcomes for South Asian older people than for older people from other ethnic groups. Older people in households with more than three other younger generations (or with six or more occupants) in the most deprived settings experienced particularly high rates of severe COVID-19, and South Asian people were over-represented in these households. Due to the large proportion of White people living alone, this group contributed nearly half of all cases of severe COVID-19 observed in White older people. Household characteristics, ethnicity and socioeconomic deprivation are important considerations, alongside individual risk factors, when considering the roll-out of COVID-19 vaccination boosters and the targeting of interventions for COVID-19 and future pandemics.

## Ethics approval

Ethical approval was granted by the Health Research Authority (REC reference 20/LO/0651) and by the London School of Hygiene and Tropical Medicine Ethics Board (reference 21863).

## Supplementary Material

dyac158_Supplementary_Data

## Data Availability

Access to the underlying identifiable and potentially re-identifiable pseudonymized electronic health record data is tightly governed by various legislative and regulatory frameworks, and restricted by best practice. The data in OpenSAFELY is drawn from general practice data across England where TPP is the data processor. TPP developers initiate an automated process to create pseudonymized records in the core OpenSAFELY database, which are copies of key structured data tables in the identifiable records. These are linked onto key external data resources that have also been pseudonymized via SHA-512 one-way hashing of NHS numbers using a shared salt. DataLab developers and principal investigators holding contracts with NHS England have access to the OpenSAFELY pseudonymized data tables as needed to develop the OpenSAFELY tools. These tools in turn enable researchers with OpenSAFELY data access agreements to write and execute code for data management and data analysis without direct access to the underlying raw pseudonymized patient data, and to review the outputs of this code. All code for the full data management pipeline—from raw data to completed results for this analysis—and for the OpenSAFELY platform as a whole is available for review at [https://github.com/opensafely/hh-classification-research].
