# Correction to: Association between household composition and severe COVID-19 outcomes in older people by ethnicity: an observational cohort study using the OpenSAFELY platform

**DOI:** 10.1093/ije/dyad041

**Published:** 2023-04-02

This is a correction to: Kevin Wing, Daniel J Grint, Rohini Mathur, Hamish P Gibbs, George Hickman, Emily Nightingale, Anna Schultze, Harriet Forbes, Vahé Nafilyan, Krishnan Bhaskaran, Elizabeth Williamson, Thomas House, Lorenzo Pellis, Emily Herrett, Nileesa Gautam, Helen J Curtis, Christopher T Rentsch, Angel Y S Wong, Brian MacKenna, Amir Mehrkar, Seb Bacon, Ian J Douglas, Stephen J W Evans, Laurie Tomlinson, Ben Goldacre, Rosalind M Eggo; on behalf of the OpenSAFELY consortium, *International Journal of Epidemiology*, dyac158, https://doi.org/10.1093/ije/dyac158.

In the Results, “For South Asian people, as for Wave 1, multigenerational living was associated with severe COVID-19 but not COVID-19 death …” should have read “For South Asian people, as for Wave 1, multigenerational living was associated with severe COVID-19 but not non-COVID-19 death …”.

The paper has been corrected.

