# Supplementary material for: Association between household composition and severe COVID-19 outcomes in older people by ethnicity: an observational cohort study using the OpenSAFELY platform
Source: Int J Epidemiol. 2022 Aug 13;51(6):1745–60. doi: 10.1093/ije/dyac158 (PMC9384728; doi:10.1093/ije/dyac158)
Supplement: dyac158_Supplementary_Data [file dyac158_supplementary_data.pdf]

Supplementary Material for
Association between household composition and severe COVID-19 outcomes by ethnicity - an observational cohort study using the OpenSAFELY platform

Supplementary material for Methods

OpenSAFELY - description of data source and information governance

Data source
All data were linked, stored and analysed securely within the OpenSAFELY-TPP platform: https://opensafely.org/. Data include pseudonymised data such as coded diagnoses, medications and physiological parameters. No free text data are included. All code is shared openly for review and re-use under MIT open license [https://github.com/opensafely/hh-classification-research]. Detailed pseudonymised patient data is potentially re-identifiable and therefore not shared.

Primary care records managed by the general practitioner (GP) software provider, TPP were linked to national health service (NHS) hospital records and office of national statistics (ONS) death data through OpenSAFELY-TPP.

Information governance and ethical approval
NHS England is the data controller for OpenSAFELY-TPP; TPP is the data processor; all study authors using OpenSAFELY have the approval of NHS England. This implementation of OpenSAFELY is hosted within the TPP environment which is accredited to the ISO 27001 information security standard and is NHS IG Toolkit compliant[1,2]

Patient data has been pseudonymised for analysis and linkage using industry standard cryptographic hashing techniques; all pseudonymised datasets transmitted for linkage onto OpenSAFELY are encrypted; access to the platform is via a virtual private network (VPN) connection, restricted to a small group of researchers; the researchers hold contracts with NHS England and only access the platform to initiate database queries and statistical models; all database activity is logged; only aggregate statistical outputs leave the platform environment following best practice for anonymisation of results such as statistical disclosure control for low cell counts.[3]

The OpenSAFELY research platform adheres to the obligations of the UK General Data Protection Regulation (GDPR) and the Data Protection Act 2018. In March 2020, the Secretary of State for Health and Social Care used powers under the UK Health Service (Control of Patient Information) Regulations 2002 (COPI) to require organisations to process confidential patient information for the purposes of protecting public health, providing healthcare services to the public and monitoring and managing the COVID-19 outbreak and incidents of exposure; this sets aside the requirement for patient consent.[4]

Taken together, these provide the legal bases to link patient datasets on the OpenSAFELY platform. GP practices, from which the primary care data are obtained, are required to share relevant health information to support the public health response to the pandemic, and have been informed of the OpenSAFELY analytics platform.

Health Research Authority (REC reference 20/LO/0651) and by the LSHTM Ethics Board (reference 21863).

Algorithm to identify households in TPP, using addresses registered in patient record
1) Get all address details for fully registered patients - Find all permanent patient addresses that were active on 01 Feb 2020 or recorded since - Combine the house name/number, remove punctuation (commas, full stops, apostrophes), double spaces and leading/trailing whitespace from house name/number and road - Replace street, lane etc with the abbreviation (street -> St, lane -> Ln, place -> Pl, avenue -> Ave, road -> Rd, close -> Cl, drive -> Dr) - Find registrations for patients that were active on or since 01 Feb 2020 (making sure the patient was alive on 01 Feb 2020) - Filter the patient addresses based on those registrations
2) Build an Address table using the distinct address fields - For each distinct (house name/number + road + post code), assign a unique Address_ID
3) Set the Address_ID on each individual patient address - Join to Address table from 2) on house name/number + road + post code
4) End addresses that started before the property was sold - Import land registry property sales data (www.gov.uk/government/statistical-data-sets/price-paid-data- downloads) - Apply the same processing as 1) to

the address fields - Set address IDs on the land registry house sales data using the same Address table from 2) joining on house name/number + road + post code - End all patient addresses at properties where the patient address start date was before the property was sold (under the assumption that in the majority of cases that means the occupants moved out) - If a patient address has an unset start date (imported data), use the registration start date for this step
5) Get the latest "active" patient address per patient - Filter to those which were active on 01 Feb 2020 or recorded since (need to do this again now we've ended some based on property sales) - Filter to the latest per patient
6) Create the HouseholdMember table - Insert a row for each patient address from 5), using the Address_ID as the Household_ID (one household per address)
7) Create the Household table - Select the distinct Address_IDs as the Household_ID into the Household table (one household per address) - Set the size based on the number of members - Use the PotentialCareHomeAddress table to set the CareHome flag - Check the address fields to identify NFA / Unknown addresses (e.g. postcode "ZZ99 ...", house name "NFA", road "Unknown", ...)

Analysis of the impact of household size (number of occupants)
In our main analysis of household composition we did not adjust for household size (number of occupants) as conceptually household size is a mediator of household composition. We also noted that not all categories of the household composition variable had a corresponding household size category (Supplementary Table S5), meaning that adjusting for household size would artificially reduce estimates downwards.

Household TPP coverage
In England, not all residents of a household are necessarily registered with the same general practice, which means it is possible that not all residents of a household would appear in the same software system. This means that the number of residents attributed to a household in the TPP register is not necessarily equal to the true total number of people in the household.
While all households in our study will have included at least one 67+ year old registered at a practice using TPP software, it is possible that some of the other people in some of the households may be registered with other practices that use software other than TPP. For these households, the calculated TPP household size (i.e. the count of records in TPP under the same household ID) will be different from the household size defined in the Master Patient Index (MPI) for the address covered by the TPP household ID. Those people who are registered to non-TPP practices will not have been counted when we created our household composition exposure variable, and this measurement error has the potential to bias results. A "TPP coverage" flag is provided for each household, which compares the TPP household size with the number of records in the Master Patient Index for the same address and is used to indicate the % of occupants of that household who are registered with general practices that use TPP.

For our main analysis, we included all households, irrespective of TPP coverage. In order to assess the impact of this design choice on our results, we performed a sensitivity analysis where we only included households with 100% TPP coverage.

Table S1: Deviations from the original study protocol

Deviation	Rationale
Our main exposure was categorised into 5 categories rather than 3 as detailed in the protocol	This change was made to facilitate analysis for a test for trend of increasing/decreasing severe COVID with increasing number of generations.
Our primary outcome in the analysis was a combined outcome of COVID death or hospitalisation. In the protocol we specified these as separate outcomes but not as a combined outcome.	This additional outcome definition was included in order to increase power to detect effects in all ethnic groups analysed.
We included a step to compare the distribution of household sizes by ethnicity with ONS figures from the 2011 census.	This was to ensure that the TPP method for assigning people to households was correct.
We added a number of ways of identifying people in care homes to the final analysis (vs only a single method in the protocol).	This change was made based on best practice for identifying care homes (Schultze et al 2021) that was published during preparation of our own analysis.
In the analysis we looked for evidence of interaction between ethnicity and any other variable, and if there were interactions found between ethnicity and any household-level variable (i.e. deprivation or housing density) presented strata for these, whereas in the protocol we only said we would stratify by deprivation.	This was to ensure we did not miss any potentially important interactions with ethnicity.

Table S2: Table showing high-level ethnicity with component ethnicity categories

High level ethnicity categories	Component categories
White	White British, White Irish, other White
South Asian	Indian, Pakistani, Bangladeshi, other South Asian
Black	African, Caribbean, other Black
Mixed	White and Asian, White and African, White and Caribbean, other mixed
Other	Chinese, all others

Table S3: Table of covariate definitions

Covariate	Description
Sex	Male or female
Age	Restricted cubic splines were used for the main analysis of each ethnicity cohort. When grouped (for descriptive reporting and when including interaction with age in the combined ethnicity cohort) age groups were: 67-69, 70-74, 75-79, 80-84, 85+.
Ethnicity	Categorised as a five-level variable based on primary care self reporting of: White, Mixed, South Asian, Black or Other
BMI	Body mass index (BMI) was ascertained within the 10 years prior to 1 Feb 2020 and recorded when the patient was over 16 years old. Grouped using categories derived from the World Health Organisation classification of BMI: underweight, normal, overweight, obese I 30-34.9; obese II 35-39.9; obese III 40+. Those with missing BMI were assigned to the normal weight category.
Smoking status	Grouped into current, former and never smokers. Missing smoking data were assigned to the never smokers category.
Index of Multiple Deprivation	Definition of deprivation using quintiles of the Index of Multiple Deprivation, an area-level composite measure of seven domains: income, employment, education, skills and training, health and disability, crime, and barriers to housing services and living environment.[6]
Region of England	Nine categories: East, East Midlands, London, North East, North West, South East, South West, West Midlands, Yorkshire & The Humber
Geographic area	ONS UTLA (Upper Tier Local Authority) - used as a stratification variable
Housing density	ONS Rural/Urban classification, divided into five categories: urban major conurbation, urban minor conurbation, urban city and town, rural town, rural village.
Total number of people in the household	Categorised as 1-2, 3-5, 6 or more.
Comorbidities	Presence of either 0, 1 or 2 or more comorbidities (see Table S3 below for further details)

Table S4: Definitions of comorbidities

Based on previous work on association of specific comorbidities with severe COVID-19,[5] we included a comorbidity variable with categories capturing whether each 67+ year old included in the study had either 0, 1 or 2+ comorbidities recorded prior to their start of follow-up. The list of conditions and codelists that were used to define the presence of comorbidity are provided below.

Condition	Codelist defining presence of comorbidity
Aplastic anaemia	https://codelists.opensafely.org/codelist/opensafely/aplastic-anaemia/
Asplenia	https://codelists.opensafely.org/codelist/opensafely/asplenia/
Asthma	https://codelists.opensafely.org/codelist/opensafely/asthma-diagnosis/<br>https://codelists.opensafely.org/codelist/opensafely/asthma-inhaler-salbutamol-medication/2020-04-15/<br>https://codelists.opensafely.org/codelist/opensafely/asthma-inhaler-steroid-medication/2020-04-15/<br>https://codelists.opensafely.org/codelist/opensafely/asthma-oral-prednisolone-medication/2020-04-27/
Bone marrow transplant	https://codelists.opensafely.org/codelist/opensafely/bone-marrow-transplant/2020-04-15/
Cancer	https://codelists.opensafely.org/codelist/opensafely/cancer-excluding-lung-and-haematological/2020-04-15/<br>https://codelists.opensafely.org/codelist/opensafely/chemotherapy-or-radiotherapy-updated/2020-04-15/<br>https://codelists.opensafely.org/codelist/opensafely/haematological-cancer/2020-04-15/<br>https://codelists.opensafely.org/codelist/opensafely/lung-cancer/2020-04-15/
Chronic cardiac disease	https://codelists.opensafely.org/codelist/opensafely/chronic-cardiac-disease/2020-04-08/
Chronic respiratory disease	https://codelists.opensafely.org/codelist/opensafely/chronic-respiratory-disease/2020-04-10/
Chronic liver disease	https://codelists.opensafely.org/codelist/opensafely/chronic-liver-disease/2020-06-02/
Dementia	https://codelists.opensafely.org/codelist/opensafely/dementia/2020-04-22/
Diabetes	https://codelists.opensafely.org/codelist/opensafely/diabetes/2020-04-15/
Chronic kidney disease	https://codelists.opensafely.org/codelist/opensafely/chronic-kidney-disease/2020-04-14/
GI bleed	https://codelists.opensafely.org/codelist/opensafely/gi-bleed-or-ulcer/2020-04-08/
HIV	https://codelists.opensafely.org/codelist/opensafely/hiv/2020-07-13/
Permanent immunosuppression	https://codelists.opensafely.org/codelist/opensafely/permanent-immunosuppression/2020-06-02/
Temporary immunosuppression	https://codelists.opensafely.org/codelist/opensafely/temporary-immunosuppression/2020-04-24/
Hypertension	https://codelists.opensafely.org/codelist/opensafely/hypertension/2020-04-28/
Stroke	https://codelists.opensafely.org/codelist/opensafely/stroke-updated/2020-06-02/
Inflammatory bowel disease	https://codelists.opensafely.org/codelist/opensafely/inflammatory-bowel-disease/2020-04-07/
Neurological conditions	https://codelists.opensafely.org/codelist/opensafely/other-neurological-conditions/2020-06-02/
Psoriasis	https://codelists.opensafely.org/codelist/opensafely/ra-sle-psoriasis/2020-04-14/
Sickle cell disease	https://codelists.opensafely.org/codelist/opensafely/sickle-cell-disease/2020-04-14/
Smoking	https://codelists.opensafely.org/codelist/opensafely/smoking-clear/2020-04-29/<br>https://codelists.opensafely.org/codelist/opensafely/smoking-unclear/2020-04-29/
Organ transplant	https://codelists.opensafely.org/codelist/opensafely/solid-organ-transplantation/2020-04-10/

Table S5: Table showing that not all categories of household composition have a corresponding household size category

Household composition category	Possible household size					
	1	2	3	4	5	6+
Multiple 67+ year olds (max 4*)	✗	✓	✓	✓	✗	✗
67+ living alone	✓	✗	✗	✗	✗	✗
67+ & 1 other generation	✗	✓	✓	✓	✓	✓
67+ & 2 other generations	✗	✗	✓	✓	✓	✓
67+ & 3 other generations	✗	✗	✗	✓	✓	✓

*largest allowable size of house with only 67+ year olds in in this study

Table S6: Categories for variable combining household size and household composition

Combined hh composition - hh size variable
Two 67+ year olds (hhsize=2)
>2 67+ year olds (hhsize=3-4)
67+ & 1 gen (hhsize=2)
67+ & 1 gen (hhsize=3-4)
67+ & 1 gen (hhsize=5+)
67+ & 2 gen (hhsize=3-4)
67+ & 2 gen (hhsize=5+)
67+ & 3 gen (hhsize=3-4)
67+ & 3 gen (hhsize=5+)

Software and Reproducibility

This analysis was delivered through the OpenSAFELY platform: codelists and code for data management and data analysis were specified using the OpenSAFELY tools; then transmitted securely to the OpenSAFELY-TPP platform within TPP's secure environment, where they were executed against local patient data; summary results were then reviewed for disclosiveness, released, and formatted for the final outputs. All code for the OpenSAFELY platform for data management, analysis and secure code execution is shared for review and re-use under open licenses at GitHub.com/OpenSAFELY. Data management and analysis was performed using Python 3.8 and Stata 16.1. Code for data management and analysis as well as codelists archived online https://github.com/opensafely/hh-classification-research.

Supplementary material for Results

Figure S1: Flow diagram of cohort with numbers excluded at different stages for wave 1 and wave 2

Wave 1

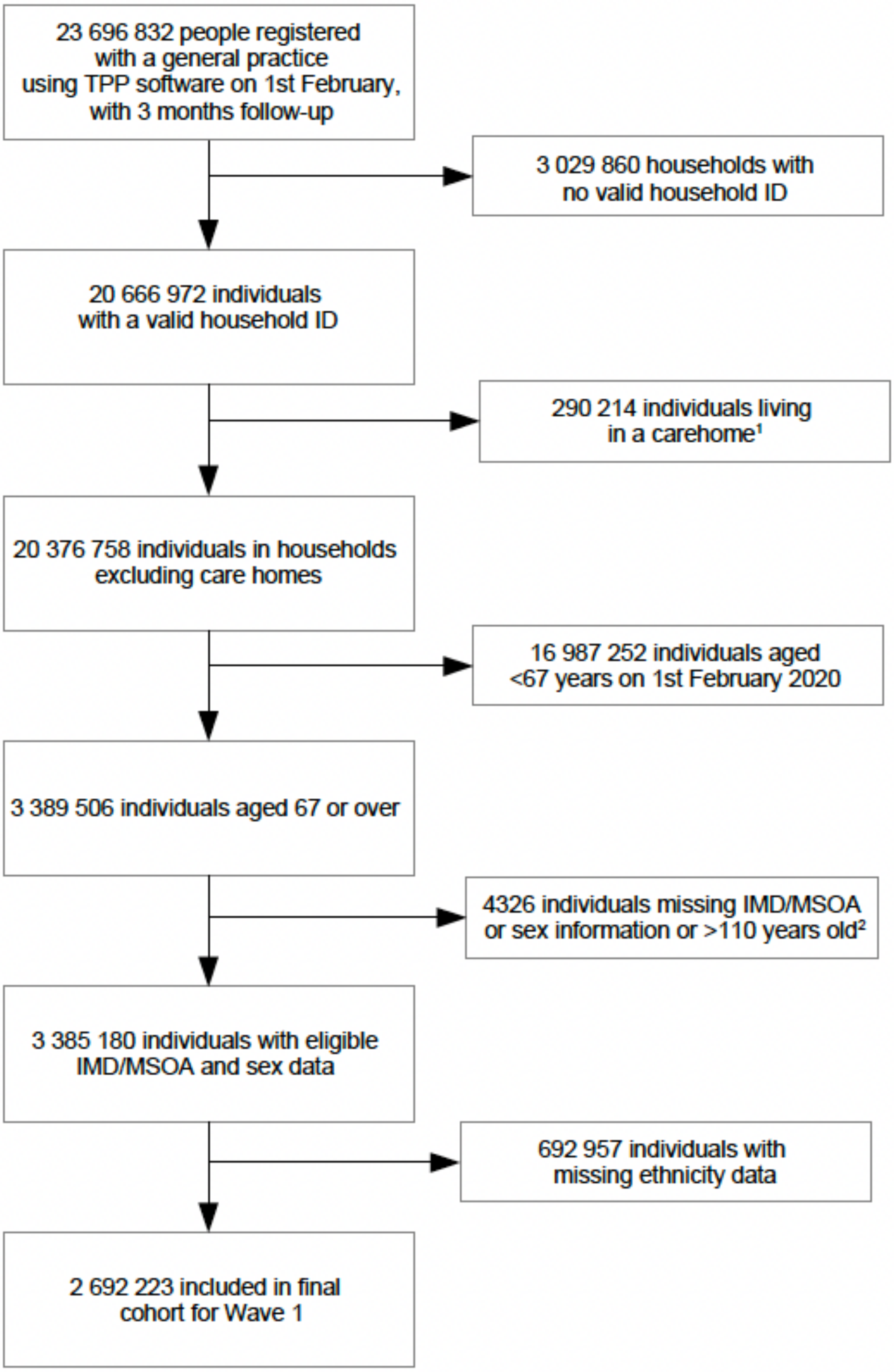

Note 1: 97 143 flagged as living in a care home, 13 999 living in a house that had an individual flagged as living in a care home, 176 701 living in a private home >12 in size, 2 371 living in a private home >4 in size with all occupants >67.
Note 2: 4 312 missing IMD/MSOA or sex information, 14 aged>110 years

Wave 2

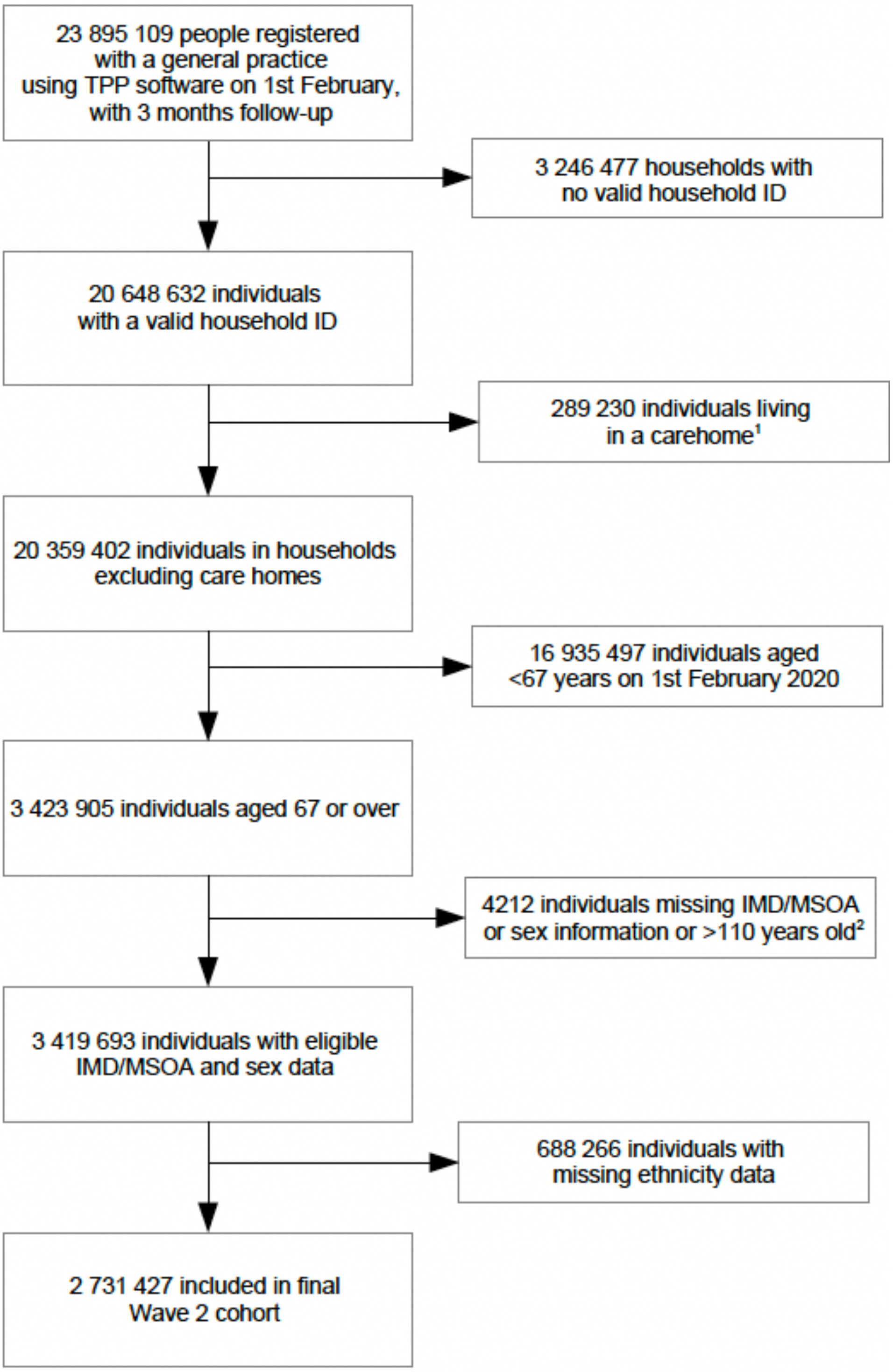

Note 1: 93 548 flagged as living in a care home, 27 420 living in a house that had an individual flagged as living in a care home, 166 723 living in a private home >12 in size, 1 539 living in a private home >4 in size with all occupants >67.
Note 2: 4 198 missing IMD/MSOA or sex information, 14 aged>110 years

Table S7: Association between household composition (number of generations in a household) and severe COVID (hospitalisation or death as a combined outcome) or non-COVID death in 67+ year olds, for separate ethnicity cohorts during the first and second waves of the pandemic in the UK, with serial adjustment

A. Death or hospitalisation from COVID-19 (combined outcome)

	Wave 1 (1st February – 31st August 2020)						Wave 2 (1st September – 31st January 2021)					
				HR (95% CI)						HR (95% CI)		
**HH composition[1]**	**N (%)**	**P-years**	**Rate[2]**	**Crude[3]**	**Age-adjusted[3]**	**Plus sex, deprivation, comorbidities clinical factors[4]**	**N (%)**	**P-years**	**Rate[2]**	**Crude[3]**	**Age-adjusted[3]**	**Plus sex, deprivation, comorbidities clinical factors[5]**
White												
Multiple 67+	1083552 (42·5)	622902	736	1	1	1	1116533 (43·2)	461106	2145	1	1	1
67+ alone	887343 (34·8)	506608	1317	1·71 (1·64-1·78)	1·33 (1·28-1·39)	1·35 (1·30-1·41)	880434 (34·1)	361731	3892	1·76 (1·71-1·81)	1·39 (1·35-1·43)	1·37 (1·33-1·41)
67+ & 1 gen	482696 (18·9)	277366	808	1·04 (0·98-1·09)	1·21 (1·15-1·27)	1·14 (1·08-1·20)	489303 (18·9)	201980	2442	1·10 (1·06-1·14)	1·29 (1·24-1·34)	1·22 (1·17-1·26)
67+ & 2 gens	82362 (3·2)	47317	845	1·06 (0·95-1·17)	1·28 (1·15-1·42)	1·22 (1·10-1·35)	84368 (3·3)	34845	2431	1·09 (1·01-1·17)	1·33 (1·24-1·43)	1·26 (1·17-1·36)
67+ & 3 gens	12309 (0·5)	7057	907	1·10 (0·86-1·40)	1·28 (1·00-1·64)	1·21 (0·94-1·55)	12756 (0·5)	5257	3291	1·44 (1·24-1·68)	1·70 (1·46-1·97)	1·61 (1·38-1·87)
South Asian												
Multiple 67+	15264 (17·5)	8741	1579	1	1	1	15969 (17·8)	6572	4200	1	1	1
67+ alone	14674 (16·8)	8366	2510	1·53 (1·22-1·91)	1·42 (1·14-1·77)	1·47 (1·18-1·84)	14745 (16·4)	6064	5607	1·31 (1·11-1·55)	1·23 (1·04-1·45)	1·22 (1·03-1·44)
67+ & 1 gen	26557 (30·5)	15195	1889	1·14 (0·92-1·41)	1·20 (0·97-1·48)	1·17 (0·95-1·45)	27605 (30·8)	11355	5161	1·19 (1·03-1·39)	1·27 (1·09-1·47)	1·22 (1·05-1·42)
67+ & 2 gens	22650 (26·0)	12946	2186	1·33 (1·07-1·64)	1·36 (1·09-1·68)	1·34 (1·08-1·66)	23136 (25·8)	9494	6362	1·46 (1·26-1·70)	1·51 (1·30-1·75)	1·45 (1·25-1·69)
67+ & 3 gens	7987 (9·2)	4560	2478	1·49 (1·15-1·92)	1·45 (1·12-1·87)	1·41 (1·09-1·83)	8198 (9·1)	3345	8520	1·94 (1·63-2·31)	1·91 (1·60-2·27)	1·76 (1·48-2·10)
Black												
Multiple 67+	4019 (15·2)	2295	2136	1	1	1	4132 (15·3)	1701	5057	1	1	1
67+ alone	9399 (35·5)	5346	2918	1·25 (0·90-1·74)	1·22 (0·88-1·70)	1·24 (0·89-1·72)	9462 (35·1)	3889	5760	1·09 (0·84-1·41)	1·07 (0·82-1·38)	1·07 (0·82-1·39)
67+ & 1 gen	7888 (29·8)	4506	2130	0·91 (0·64-1·30)	0·95 (0·67-1·36)	1·01 (0·71-1·44)	8050 (29·9)	3315	4194	0·80 (0·60-1·05)	0·84 (0·63-1·11)	0·88 (0·66-1·17)
67+ & 2 gens	4017 (15·2)	2305	2213	0·97 (0·65-1·46)	1·09 (0·73-1·62)	1·27 (0·85-1·90)	4119 (15·3)	1700	3354	0·64 (0·45-0·90)	0·72 (0·51-1·01)	0·83 (0·59-1·17)
67+ & 3 gens	1155 (4·4)	661	1966	0·85 (0·46-1·58)	0·94 (0·51-1·74)	1·16 (0·62-2·14)	1185 (4·4)	490	4281	0·82 (0·50-1·32)	0·90 (0·56-1·47)	1·11 (0·69-1·81)
Mixed												
Multiple 67+	2027 (21·6)	1162	1290	1	1	1	2117 (21·8)	871	3903	1	1	1
67+ alone	3129 (33·3)	1784	1962	1·36 (0·71-2·63)	1·35 (0·70-2·61)	1·29 (0·67-2·49)	3188 (32·9)	1310	4198	1·02 (0·66-1·57)	0·97 (0·62-1·50)	0·90 (0·57-1·42)
67+ & 1 gen	2711 (28·9)	1554	1480	1·01 (0·50-2·03)	1·16 (0·58-2·32)	1·16 (0·58-2·32)	2832 (29·2)	1169	2824	0·68 (0·42-1·10)	0·70 (0·44-1·14)	0·70 (0·43-1·15)
67+ & 2 gens	1209 (12·9)	696	1437	0·96 (0·41-2·23)	1·12 (0·48-2·60)	1·24 (0·53-2·87)	1234 (12·7)	509	4318	1·01 (0·59-1·74)	1·09 (0·63-1·86)	1·12 (0·65-1·92)
67+ & 3 gens	318 (3·4)	184	0	0·00 (0·00-0·00)	0·00 (0·00-0·00)	0·00 (0·00-0·00)	325 (3·4)	134	6736	1·53 (0·73-3·21)	1·58 (0·76-3·30)	1·62 (0·77-3·41)
Other												
Multiple 67+	4581 (21·9)	2635	721	1	1	1	4807 (22·1)	1984	3478	1	1	1
67+ alone	6125 (29·2)	3505	1541	1·89 (1·10-3·25)	1·72 (1·00-2·97)	1·72 (0·99-2·97)	6210 (28·6)	2559	4299	1·18 (0·86-1·62)	1·12 (0·82-1·54)	1·10 (0·80-1·51)
67+ & 1 gen	6185 (29·5)	3558	1237	1·54 (0·88-2·69)	1·65 (0·94-2·89)	1·66 (0·95-2·91)	6486 (29·8)	2682	2908	0·79 (0·56-1·11)	0·86 (0·61-1·21)	0·84 (0·60-1·19)
67+ & 2 gens	3295 (15·7)	REDACT	REDACT	REDACT	1·75 (0·94-3·26)	1·88 (1·01-3·50)	3421 (15·7)	1417	3034	0·82 (0·56-1·21)	0·90 (0·61-1·33)	0·96 (0·65-1·42)
67+ & 3 gens	771 (3·7)	REDACT	REDACT	REDACT	0·87 (0·26-2·99)	0·89 (0·26-3·07)	812 (3·7)	335	3876	1·03 (0·54-1·94)	1·09 (0·58-2·07)	1·12 (0·59-2·13)

Note 1: Household composition in terms of number of distinct generations that each 67+ year old in the study cohort is living with (considering the following distinct generations: 0-17 year olds, 18-29 year olds, 30-66 year olds, 67+ year olds) i.e. 67+ & 1=67+ year old's houshold includes one other generation, 67+ & 2=67+ year old's houshold includes two other generations. Note 2: Rate per 100 000 person years. Note 3: Stratified by location (upper tier local authority level - UTLA). Note 4: Stratified by location (UTLA) and adjusted for sex, number of comorbidities, categories of housing density (rural or urban setting), smoking status, socio-economic status and includng an interaction between ethnicity and age (as well as the interaction between household composition and ethnicity presented here). Note 5: Stratified on UTLA and adjusted for: sex, smoking, housing density and number of comorbidities and including interactions between ethnicity and: IMD, age and obesity (as well as the interaction with household composition presented here).

Table S8: Results of statistical tests for interaction between (1) ethnicity and household composition and (2) other variables for which there was evidence of an interaction with ethnicity (for the severe COVID-19 outcome)

Variable	p (LRT for interaction)
Wave 1	
Household composition	0.696
Age	<0.0001
Wave 2	
Household composition	0.003
Age	<0.0001
Deprivation status (index of multiple deprivation - IMD)	0.006
Obesity	<0.0001

Table S9: Analysis of how the association between household compostion and severe COVID-19 varies by household size (number of occupants) via cross tabulation of results from a combined household size – household composition variable for White and South Asian ethnicities for Wave 2

Wave 2 (1st September – 31st January 2021)

**Combined household composition/household size exposure variable - multivariable adjusted HR[1] (95% CI)**	
**White**	
Two 67+ year olds (hhsize[2]=2)	1
>2 67+ year olds (hhsize=3-4)	1·52 (1·30-1·78)
67+ & 1 gen (hhsize=2)	1·19 (1·14-1·25)
67+ & 1 gen (hhsize=3-4)	1·23 (1·17-1·29)
67+ & 1 gen (hhsize=5+)	2·10 (1·74-2·54)
67+ & 2 gen (hhsize=3-4)	1·27 (1·16-1·39)
67+ & 2 gen (hhsize=5+)	1·27 (1·13-1·42)
67+ & 3 gen (hhsize=3-4)	1·54 (1·03-2·30)
67+ & 3 gen (hhsize=5+)	1·62 (1·38-1·91)

Cross-tabulation of multivariable adjusted results for the household composition/household size exposure variable (household composition rows, household size columns) — White

	**2**	**3-4**	**5+**
**67+ only**	1	1·49 (1·27-1·75)	
**67+ & 1 gen[3]**	1·18 (1·13-1·24)	1·23 (1·17-1·29)	2·08 (1·72-2·52)
**67+ & 2 gen**		1·25 (1·14-1·37)	1·24 (1·10-1·40)
**67+ & 3 gen**		1·44 (0·94-2·19)	1·57 (1·33-1·85)

**Combined household composition/household size exposure variable - multivariable adjusted HR[1] (95% CI)**	
**South Asian**	
Two 67+ year olds (hhsize=2)	1
>2 67+ year olds (hhsize=3-4)	0·77 (0·41-1·46)
67+ & 1 gen (hhsize=2)	1·21 (1·00-1·47)
67+ & 1 gen (hhsize=3-4)	1·15 (0·97-1·37)
67+ & 1 gen (hhsize=5+)	1·44 (1·08-1·92)
67+ & 2 gen (hhsize=3-4)	1·03 (0·82-1·29)
67+ & 2 gen (hhsize=5+)	1·56 (1·33-1·83)
67+ & 3 gen (hhsize=3-4)	1·34 (0·66-2·72)
67+ & 3 gen (hhsize=5+)	1·72 (1·44-2·06)

Cross-tabulation of multivariable adjusted results for the household composition/household size exposure variable (household composition rows, household size columns) — South Asian

	**2**	**3-4**	**5+**
**67+ only**	1	0·77 (0·41-1·46)	
**67+ & 1 gen**	1·21 (1·00-1·47)	1·15 (0·97-1·37)	1·44 (1·08-1·92)
**67+ & 2 gen**		1·03 (0·82-1·29)	1·56 (1·33-1·83)
**67+ & 3 gen**		1·34 (0·66-2·72)	1·72 (1·44-2·06)

Note 1: Stratified on UTLA and adjusted for: sex, smoking, housing density and number of comorbidities and including interactions between ethnicity and: IMD, age and obesity (as well as the interaction between ethnicity and the combined household composition/size variable presented here). Note 2: Household size (number of occupants in the house). Note 3: p (test-for-trend)=0·003.

Results for the separate severe COVID-19 outcomes (death and hospitalisation)
Results for analysis of the separate severe COVID-19 outcomes (death and hospitalisation) for wave 1 and wave 2 are provided in Figure S2. Associations between household composition and both of the component outcomes were generally similar to the association between household composition and the (combined) severe COVID-19 outcome for all ethnicities, with the most notable differences for White and South Asian 67+ for the death due to COVID-19 outcome in wave 2. In comparison to the White severe COVID-19 analysis, there was no trend for increasing hazard of the outcome with increasing multigenerational living for COVID-19 death (and all HRs were lower), while for South Asian people there was a steeper trend and larger HRs across the multigenerational categories.

Figure S2. Association between household composition and (1) hospitalisation due to COVID-19 and (2) death due to COVID-19 by ethnicity for wave 1 and wave 2 of the pandemic in England

Wave 1 models stratified by location (UTLA) and adjusted for sex, number of comorbidities, categories of housing density (rural or urban setting), smoking status, socio-economic status and including an interaction between ethnicity and age (as well as the interaction between household composition and ethnicity presented here). Wave 2 models stratified on UTLA and adjusted for: sex, smoking, housing density and number of comorbidities and including interactions between ethnicity and: IMD, age and obesity (as well as the interaction with household composition presented here).

Wave 1

Admitted to hospital due to COVID-19

Ethnicity and hh composition	N	Events	HR (95% CI)
White			
Multiple 67+ year olds	1083552	4202 (.4%)	1.00 (1.00, 1.00)
67+ living alone	887343	6018 (.7%)	1.35 (1.29, 1.41)
67+ & 1 other gen	482696	2069 (.4%)	1.14 (1.08, 1.21)
67+ & 2 other gens	82362	372 (.5%)	1.23 (1.10, 1.37)
67+ & 3 other gens	12309	60 (.5%)	1.23 (0.95, 1.58)
South Asian			
Multiple 67+ year olds	15264	128 (.8%)	1.00 (1.00, 1.00)
67+ living alone	14674	189 (1.3%)	1.43 (1.13, 1.80)
67+ & 1 other gen	26557	268 (1%)	1.18 (0.95, 1.47)
67+ & 2 other gens	22650	263 (1.2%)	1.34 (1.07, 1.67)
67+ & 3 other gens	7987	101 (1.3%)	1.35 (1.04, 1.77)
Black			
Multiple 67+ year olds	4019	47 (1.2%)	1.00 (1.00, 1.00)
67+ living alone	9399	143 (1.5%)	1.20 (0.85, 1.68)
67+ & 1 other gen	7888	86 (1.1%)	0.95 (0.66, 1.38)
67+ & 2 other gens	4017	46 (1.1%)	1.22 (0.80, 1.85)
67+ & 3 other gens	1155	13 (1.1%)	1.23 (0.66, 2.28)
Mixed			
Multiple 67+ year olds	2027	13 (.6%)	1.00 (1.00, 1.00)
67+ living alone	3129	32 (1%)	1.36 (0.67, 2.77)
67+ & 1 other gen	2711	19 (.7%)	1.09 (0.51, 2.32)
67+ & 2 other gens	1209	8 (.7%)	1.12 (0.44, 2.82)
Other			
Multiple 67+ year olds	4581	REDACT (REDACT%)	1.00 (1.00, 1.00)
67+ living alone	6125	48 (.8%)	1.63 (0.93, 2.88)
67+ & 1 other gen	6185	42 (.7%)	1.68 (0.94, 2.98)
67+ & 2 other gens	3295	20 (.6%)	1.65 (0.85, 3.19)
67+ & 3 other gens	771	REDACT (REDACT%)	0.94 (0.27, 3.25)

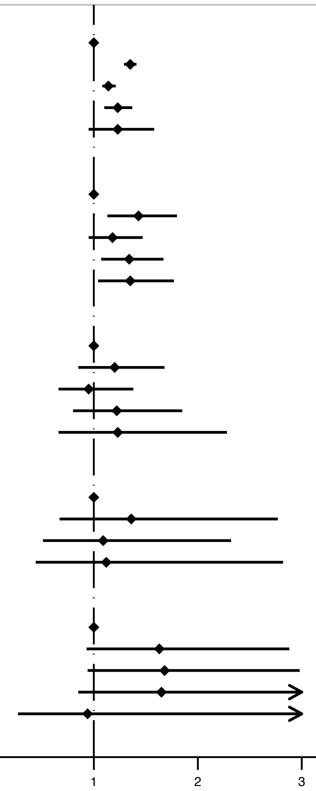

Death due to COVID-19

Ethnicity and hh composition	N	Events	HR (95% CI)
White			
Multiple 67+ year olds	1083552	1972 (.2%)	1.00 (1.00, 1.00)
67+ living alone	887343	2877 (.3%)	1.31 (1.23, 1.39)
67+ & 1 other gen	482696	861 (.2%)	1.09 (1.01, 1.19)
67+ & 2 other gens	82362	140 (.2%)	1.08 (0.91, 1.29)
67+ & 3 other gens	12309	21 (.2%)	1.00 (0.65, 1.54)
South Asian			
Multiple 67+ year olds	15264	62 (.4%)	1.00 (1.00, 1.00)
67+ living alone	14674	100 (.7%)	1.62 (1.17, 2.24)
67+ & 1 other gen	26557	129 (.5%)	1.26 (0.92, 1.71)
67+ & 2 other gens	22650	138 (.6%)	1.54 (1.13, 2.09)
67+ & 3 other gens	7987	48 (.6%)	1.41 (0.96, 2.07)
Black			
Multiple 67+ year olds	4019	23 (.6%)	1.00 (1.00, 1.00)
67+ living alone	9399	67 (.7%)	1.15 (0.72, 1.85)
67+ & 1 other gen	7888	37 (.5%)	0.85 (0.51, 1.43)
67+ & 2 other gens	4017	REDACT (-%)	1.15 (0.64, 2.08)
67+ & 3 other gens	1155	REDACT (-%)	0.40 (0.10, 1.70)
Mixed			
Multiple 67+ year olds	2027	REDACT (-%)	1.00 (1.00, 1.00)
67+ living alone	3129	12 (.4%)	0.72 (0.30, 1.75)
67+ & 1 other gen	2711	12 (.4%)	0.99 (0.41, 2.39)
67+ & 2 other gens	1209	REDACT (-%)	0.60 (0.16, 2.30)
Other			
Multiple 67+ year olds	4581	9 (.2%)	1.00 (1.00, 1.00)
67+ living alone	6125	25 (.4%)	1.66 (0.72, 3.83)
67+ & 1 other gen	6185	13 (.2%)	1.07 (0.43, 2.68)
67+ & 2 other gens	3295	11 (.3%)	1.88 (0.72, 4.90)

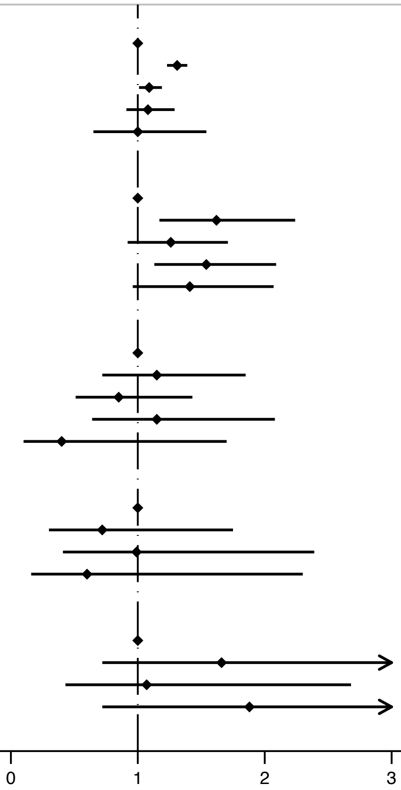

Wave 2

Admitted to hospital due to COVID-19

Ethnicity and hh composition	N	Events	HR (95% CI)
**White**			
Multiple 67+	1116533	9466 (.8%)	1.00 (1.00, 1.00)
67+ living alone	880434	13414 (1.5%)	1.37 (1.33, 1.41)
67+ & 1 other gen	489303	4738 (1%)	1.22 (1.18, 1.27)
67+ & 2 other gens	84368	817 (1%)	1.27 (1.18, 1.37)
67+ & 3 other gens	12756	169 (1.3%)	1.64 (1.40, 1.91)
**South Asian**			
Multiple 67+	15969	268 (1.7%)	1.00 (1.00, 1.00)
67+ living alone	14745	319 (2.2%)	1.18 (1.00, 1.40)
67+ & 1 other gen	27605	546 (2%)	1.17 (1.01, 1.37)
67+ & 2 other gens	23136	571 (2.5%)	1.42 (1.21, 1.65)
67+ & 3 other gens	8198	273 (3.3%)	1.74 (1.46, 2.09)
**Black**			
Multiple 67+	4132	81 (2%)	1.00 (1.00, 1.00)
67+ living alone	9462	218 (2.3%)	1.10 (0.84, 1.45)
67+ & 1 other gen	8050	133 (1.7%)	0.89 (0.67, 1.19)
67+ & 2 other gens	4119	56 (1.4%)	0.86 (0.61, 1.23)
67+ & 3 other gens	1185	19 (1.6%)	1.07 (0.64, 1.78)
**Mixed**			
Multiple 67+	2117	30 (1.4%)	1.00 (1.00, 1.00)
67+ living alone	3188	52 (1.6%)	0.97 (0.60, 1.56)
67+ & 1 other gen	2832	32 (1.1%)	0.78 (0.47, 1.30)
67+ & 2 other gens	1234	20 (1.6%)	1.15 (0.65, 2.05)
**Other**			
Multiple 67+	4807	67 (1.4%)	1.00 (1.00, 1.00)
67+ living alone	6210	102 (1.6%)	1.04 (0.75, 1.44)
67+ & 1 other gen	6486	75 (1.2%)	0.83 (0.59, 1.18)
67+ & 2 other gens	3421	43 (1.3%)	0.99 (0.67, 1.47)
67+ & 3 other gens	812	12 (1.5%)	1.05 (0.54, 2.05)

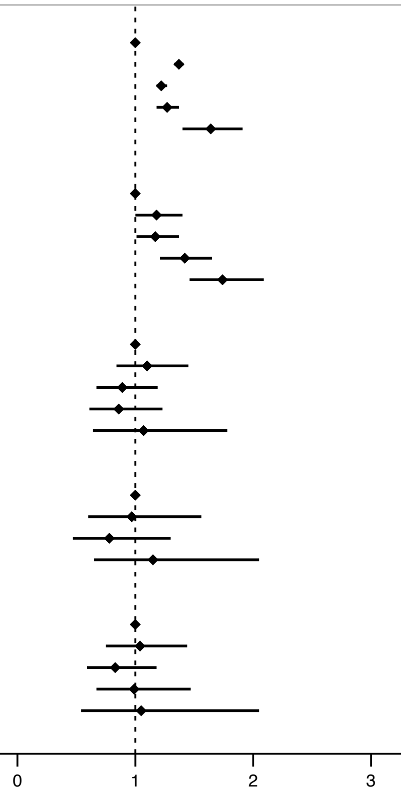

Death due to COVID-19

Ethnicity and hh composition	N	Events	HR (95% CI)
**White**			
Multiple 67+	1116533	3371 (.3%)	1.00 (1.00, 1.00)
67+ living alone	880434	4654 (.5%)	1.28 (1.22, 1.34)
67+ & 1 other gen	489303	1506 (.3%)	1.18 (1.11, 1.26)
67+ & 2 other gens	84368	220 (.3%)	1.06 (0.92, 1.22)
67+ & 3 other gens	12756	46 (.4%)	1.37 (1.02, 1.83)
**South Asian**			
Multiple 67+	15969	93 (.6%)	1.00 (1.00, 1.00)
67+ living alone	14745	118 (.8%)	1.29 (0.97, 1.70)
67+ & 1 other gen	27605	211 (.8%)	1.37 (1.07, 1.77)
67+ & 2 other gens	23136	207 (.9%)	1.53 (1.19, 1.97)
67+ & 3 other gens	8198	115 (1.4%)	2.12 (1.60, 2.82)
**Black**			
Multiple 67+	4132	36 (.9%)	1.00 (1.00, 1.00)
67+ living alone	9462	74 (.8%)	0.92 (0.61, 1.40)
67+ & 1 other gen	8050	44 (.5%)	0.74 (0.47, 1.16)
67+ & 2 other gens	4119	21 (.5%)	0.85 (0.49, 1.48)
67+ & 3 other gens	1185	11 (.9%)	1.63 (0.82, 3.23)
**Mixed**			
Multiple 67+	2117	14 (.7%)	1.00 (1.00, 1.00)
67+ living alone	3188	24 (.8%)	0.97 (0.49, 1.92)
67+ & 1 other gen	2832	10 (.4%)	0.56 (0.25, 1.26)
67+ & 2 other gens	1234	REDACT (0%)	1.35 (0.60, 3.05)
**Other**			
Multiple 67+	4807	21 (.4%)	1.00 (1.00, 1.00)
67+ living alone	6210	40 (.6%)	1.40 (0.81, 2.44)
67+ & 1 other gen	6486	15 (.2%)	0.62 (0.32, 1.22)
67+ & 2 other gens	3421	REDACT (0%)	0.70 (0.30, 1.60)
67+ & 3 other gens	812	REDACT (0%)	1.79 (0.66, 4.89)

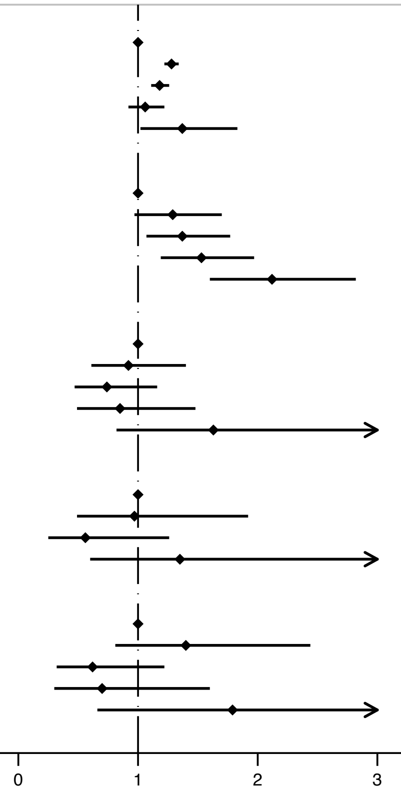

Figure S3. Association between household composition and severe COVID-19 (death or hospitalisation due to COVID-19) by the component White and South Asian ethnicity categories for wave 2 of the pandemic in England

Model stratified on UTLA and adjusted for: sex, smoking, housing density and number of comorbidities and including interactions between ethnicity and: IMD, age and obesity (as well as the interaction with household composition presented here).

Wave 2: Death or hospital admission due to COVID-19

Ethnicity and hh composition	Denominator	Events	HR (95% CI)
**British**			
Multiple 67+ year olds	1209019	10556 (.9%)	1.00 (1.00, 1.00)
67+ living alone	831314	13325 (1.6%)	1.36 (1.32, 1.40)
67+ & 1 other gen	326939	3546 (1.1%)	1.25 (1.20, 1.30)
67+ & 2 other gens	63308	666 (1.1%)	1.27 (1.17, 1.38)
67+ & 3 other gens	9562	130 (1.4%)	1.57 (1.32, 1.87)
**Irish**			
Multiple 67+ year olds	8758	96 (1.1%)	1.00 (1.00, 1.00)
67+ living alone	8971	158 (1.8%)	1.28 (0.98, 1.65)
67+ & 1 other gen	4616	49 (1.1%)	0.90 (0.64, 1.28)
67+ & 2 other gens	REDACT	REDACT (REDACT%)	0.67 (0.31, 1.43)
67+ & 3 other gens	REDACT	REDACT (REDACT%)	1.93 (0.71, 5.21)
**Other White**			
Multiple 67+ year olds	52091	497 (1%)	1.00 (1.00, 1.00)
67+ living alone	43321	675 (1.6%)	1.29 (1.14, 1.45)
67+ & 1 other gen	21818	268 (1.2%)	1.30 (1.11, 1.51)
67+ & 2 other gens	9604	99 (1%)	1.33 (1.06, 1.66)
67+ & 3 other gens	2463	28 (1.1%)	1.44 (0.98, 2.10)
**Indian**			
Multiple 67+ year olds	13119	207 (1.6%)	1.00 (1.00, 1.00)
67+ living alone	8059	181 (2.2%)	1.29 (1.05, 1.59)
67+ & 1 other gen	14151	249 (1.8%)	1.14 (0.94, 1.38)
67+ & 2 other gens	10807	230 (2.1%)	1.40 (1.15, 1.70)
67+ & 3 other gens	2844	67 (2.4%)	1.51 (1.13, 2.02)
**Pakistani**			
Multiple 67+ year olds	2284	74 (3.2%)	1.00 (1.00, 1.00)
67+ living alone	2576	78 (3%)	0.92 (0.67, 1.28)
67+ & 1 other gen	5268	165 (3.1%)	1.01 (0.76, 1.34)
67+ & 2 other gens	6907	235 (3.4%)	1.11 (0.85, 1.46)
67+ & 3 other gens	3566	168 (4.7%)	1.49 (1.12, 1.98)
**Bangladeshi**			
Multiple 67+ year olds	256	12 (4.7%)	1.00 (1.00, 1.00)
67+ living alone	440	14 (3.2%)	0.64 (0.27, 1.53)
67+ & 1 other gen	760	23 (3%)	0.65 (0.30, 1.43)
67+ & 2 other gens	1128	57 (5.1%)	1.07 (0.52, 2.20)
67+ & 3 other gens	667	20 (3%)	0.61 (0.27, 1.36)
**Other South Asian**			
Multiple 67+ year olds	4170	62 (1.5%)	1.00 (1.00, 1.00)
67+ living alone	3691	68 (1.8%)	1.17 (0.82, 1.67)
67+ & 1 other gen	4542	90 (2%)	1.28 (0.92, 1.80)
67+ & 2 other gens	3500	74 (2.1%)	1.41 (0.99, 2.00)
67+ & 3 other gens	1060	25 (2.4%)	1.42 (0.88, 2.28)

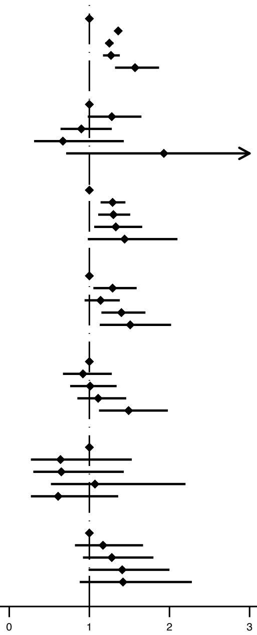

Analysis of the impact of household size

Cross-tabulation of household composition against household size (Table S9 - panel 2) showed that increasing household size within categories of household composition (and vice versa) did not correspond to an increasing hazard of severe COVID-19 for either White or South Asian older people (apart from a trend in increasing hazards with increasing household size within the White 67+ & 1 generation). Of note was that for White 67+, across the combined household composition-household size exposure variable, two of the three highest HRs were within the largest household size category (5+) and for South Asian over 67+ year olds all three of the highest HRs were within the largest household size category.

Table S10: Multivariable-adjusted association between deprivation and (1) severe COVID (hospitalisation or death as a combined outcome) and (2) death from a cause other than COVID-19 by ethnicity in 67+ year olds for Wave 2

	Wave 2 (1st September – 31st January 2021)				
	Multivariable HR[1] (95% CI)				
	**White**	**South Asian**	**Black**	**Mixed**	**Other**
**Severe COVID-19**					
IMD (p test for interaction=0·006[2])					
1 (affluent)	1	1	1	1	1
2	1·13 (1·09-1·17)	1·53 (1·21-1·94)	1·80 (1·09-2·99)	1·00 (0·52-1·95)	1·05 (0·69-1·58)
3	1·25 (1·21-1·30)	1·67 (1·34-2·08)	1·73 (1·06-2·80)	1·30 (0·71-2·38)	1·09 (0·73-1·63)
4	1·39 (1·34-1·45)	2·00 (1·63-2·47)	1·48 (0·92-2·39)	1·30 (0·71-2·39)	1·17 (0·79-1·73)
5 (deprived)	1·65 (1·58-1·72)	2·46 (2·00-3·03)	1·76 (1·10-2·80)	2·07 (1·15-3·73)	1·30 (0·88-1·93)
**Death from a cause other than COVID-19**					
IMD (p test for interaction=0·610[2])					
1 (affluent)	1	1	1	1	1
2	1·07 (1·03-1·11)	1·09 (0·81-1·47)	0·78 (0·43-1·42)	0·56 (0·24-1·30)	1·14 (0·67-1·93)
3	1·15 (1·11-1·19)	1·24 (0·95-1·63)	0·96 (0·56-1·63)	1·12 (0·57-2·19)	0·96 (0·56-1·64)
4	1·22 (1·18-1·27)	1·28 (0·99-1·66)	0·84 (0·51-1·41)	1·16 (0·60-2·23)	1·17 (0·71-1·94)
5 (deprived)	1·34 (1·29-1·40)	1·27 (0·97-1·65)	1·25 (0·77-2·03)	1·55 (0·83-2·89)	1·41 (0·85-2·33)

Note 1: Stratified on UTLA and adjusted for: sex, smoking, housing density and number of comorbidities and including interactions between ethnicity and: household composition, age and obesity (as well as the interaction between ethnicity and IMD presented here). Note 2: LRT test

Table S11: Results of senstivitiy analyses (for the death or hospitalisation from COVID-19 combined outcome) relating to (1) including a 5 year buffer between the 67+ year old generation and the next youngest generation (2) only including people who lived in households with 100% TPP coverage (3) using multiple imputation to account for missing ethnicity and (4) a complete records analysis for BMI and smoking.

	Wave 2 (1st September – 31st January 2021) – MV HR (95% CI)				
		Sensitivity analyses			
HH composition	Main MV adjusted analysis results (for reference)	5 year buffer	100% TPP coverage	Missing ethnicity multiply imputed	Complete records BMI and smoking
White					
Multiple 67+	1	1	1	1	1
67+ alone	1·37 (1·33-1·41)	1·36 (1·32-1·39)	1·37 (1·33-1·41)	1·52 (1·48-1·56)	1·39 (1·33-1·45)
67+ & 1 gen	1·22 (1·17-1·26)	1·24 (1·20-1·29)	1·25 (1·20-1·30)	1.21 (1.18-1.25)	1·29 (1·21-1·36)
67+ & 2 gens	1·26 (1·17-1·36)	1·27 (1·18-1·37)	1·27 (1·17-1·38)	1.21 (1.13-1.29)	1·44 (1·29-1·62)
67+ & 3 gens	1·61 (1·38-1·87)	1·55 (1·33-1·81)	1·46 (1·23-1·74)	1.42 (1.23-1.64)	1·75 (1·37-2·24)
South Asian					
Multiple 67+	1	1	1	1	1
67+ alone	1·22 (1·03-1·44)	1·17 (1·00-1·36)	1·17 (0·99-1·38)	1.38 (1.19-1.61)	1·15 (0·91-1·44)
67+ & 1 gen	1·22 (1·05-1·42)	1·15 (1·00-1·33)	1·12 (0·96-1·31)	1.25 (1.09-1.44)	1·11 (0·90-1·36)
67+ & 2 gens	1·45 (1·25-1·69)	1·41 (1·23-1·62)	1·45 (1·25-1·69)	1.47 (1.28-1.69)	1·40 (1·14-1·72)
67+ & 3 gens	1·76 (1·48-2·10)	1·66 (1·41-1·96)	1·69 (1·41-2·03)	1.76 (1.49-2.06)	1·72 (1·35-2·18)
Black					
Multiple 67+	1	1	1	1	1
67+ alone	1·07 (0·82-1·39)	1·12 (0·88-1·44)	1·16 (0·90-1·51)	1.27 (1.01-1.60)	1·21 (0·85-1·72)
67+ & 1 gen	0·88 (0·66-1·17)	0·94 (0·72-1·24)	0·89 (0·67-1·19)	0.88 (0.69-1.13)	0·93 (0·62-1·37)
67+ & 2 gens	0·83 (0·59-1·17)	0·84 (0·60-1·18)	0·86 (0·60-1·25)	0.74 (0.54-1.02)	0·84 (0·50-1·41)
67+ & 3 gens	1·11 (0·69-1·81)	1·14 (0·70-1·84)	1·11 (0·63-1·96)	1.06 (0.68-1.65)	1·29 (0·60-2·76)
Mixed					
Multiple 67+	1	1	1	1	1
67+ alone	0·90 (0·57-1·42)	1·07 (0·69-1·66)	1·21 (0·75-1·93)	0.74 (0.52-1.05)	1·10 (0·56-2·17)
67+ & 1 gen	0·70 (0·43-1·15)	0·94 (0·58-1·53)	0·80 (0·46-1·41)	0.69 (0.48-1.00)	0·84 (0·39-1·79)
67+ & 2 gens	1·12 (0·65-1·92)	1·35 (0·79-2·30)	1·21 (0·64-2·27)	1.04 (0.69-1.58)	1·67 (0·74-3·77)
67+ & 3 gens	1·62 (0·77-3·41)	1·71 (0·79-3·67)	1·31 (0·46-3·73)	1.20 (0.64-2.24)	2·56 (0·85-7·65)
Other					
Multiple 67+	1	1	1	1	1
67+ alone	1·10 (0·80-1·51)	1·15 (0·85-1·55)	1·22 (0·88-1·68)	0.90 (0.69-1.17)	1·05 (0·68-1·63)
67+ & 1 gen	0·84 (0·60-1·19)	0·88 (0·63-1·23)	0·92 (0·64-1·33)	0.66 (0.49-0.88)	0·98 (0·61-1·58)
67+ & 2 gens	0·96 (0·65-1·42)	0·99 (0·68-1·46)	1·06 (0·69-1·62)	0.97 (0.71-1.32)	1·16 (0·66-2·03)
67+ & 3 gens	1·12 (0·59-2·13)	1·18 (0·63-2·22)	1·33 (0·67-2·61)	1.32 (0.83-2.10)	1·05 (0·35-3·15)

Table S12: Comparison of OpenSAFELY (TPP) classification of household composition with 2011 Census data

	White		South Asian		Black		Mixed		Other	
	Census	TPP	Census	TPP	Census	TPP	Census	TPP	Census	TPP
Only 67+ (alone or with other 67+)	78%	77%	33%	34%	57%	51%	69%	55%	47%	51%
67+ with 1 generation	19%	19%	26%	31%	29%	30%	23%	29%	29%	29%
67+ with 2 generation	2%	3%	28%	26%	12%	15%	6%	19%	19%	16%
67+ with 3 generation	0%	1%	13%	9%	3%	4%	1%	6%	6%	4%

References for supplementary material

1. BETA -Data Security Standards - NHS Digital. NHS Digital. https://digital.nhs.uk/about-nhs-digital/our-work/nhs-digital-data-and-technology-standards/framework/beta---data-security-standards.
2. Data Security and Protection Toolkit - NHS Digital. NHS Digital. https://digital.nhs.uk/data-and-information/looking-after-information/data-security-and-information-governance/data-security-and-protection-toolkit.
3. ISB1523: Anonymisation Standard for Publishing Health and Social Care Data - NHS Digital. NHS Digital. https://digital.nhs.uk/data-and-information/information-standards/information-standards-and-data-collections-including-extractions/publications-and-notifications/standards-and-collections/isb1523-anonymisation-standard-for-publishing-health-and-social-care-data (accessed 11/07/2021).
4. Secretary of State for Health and Social Care - UK Government. Coronavirus (COVID-19): notification to organisations to share information. 2020. https://web.archive.org/web/20200421171727/https://www.gov.uk/government/publications/coronavirus-covid-19-notification-of-data-controllers-to-share-information (accessed 11/07/2021).
5. Williamson, EJ, Walker AJ, Bhaskaran K, *et al.* OpenSAFELY: factors associated with COVID-19 death in 17 million patients. *Nature 2020*; published online July 8 2020. DOI:10.1038/s41586-020-2521-4
6. Ministry of Housing, Communities & Local Government. English indices of deprivation 2015: Index of Multiple Deprivation. Sept 30, 2015. https://www.gov.uk/government/statistics/english- indices-of-deprivation-2015 (accessed August 2, 2021).
